# Lectin Receptor-Like Protein Kinase OsNRFG6 is Required for Embryo Sac Development and Fertilization in Neo-Tetraploid Rice

**DOI:** 10.1186/s12284-024-00720-0

**Published:** 2024-06-25

**Authors:** Chongchong Zhao, Qihang Li, Qi Ge, Rou Chen, Hang Yu, Jinwen Wu, Xiangdong Liu, Zijun Lu

**Affiliations:** 1 State Key Laboratory for Conservation and Utilization of Subtropical Agro-Bioresources, Guangdong Laboratory for Lingnan Modern Agriculture, South China Agricultural University, Guangzhou, 510642 China; 2 Rice Research Institute, Guangdong Academy of Agricultural Sciences, Guangzhou, 510640 China; 3 Guangdong Provincial Key Laboratory of Plant Molecular Breeding, South China Agricultural University, Guangzhou, 510642 China; 4 Guangdong Base Bank for Lingnan Rice Germplasm Resources, College of Agriculture, South China Agricultural University, Guangzhou, 510642 China

**Keywords:** Lectin receptor-like Kinase (LecRLK), Neo-tetraploid rice, Fertility, Embryo sac, Double fertilization

## Abstract

Great yield-enhancing prospects of autotetraploid rice was restricted by various polyploidy-induced reproductive dysfunction. To surmount these challenges, our group has generated a series of valuable fertile tetraploid lines (denoted as neo-tetraploid rice) through 20-year efforts. With this context, a G-type lectin receptor-like kinase, OsNRFG6, was identified as a pivotal factor associated with reproductive regulation in neo-tetraploid rice. Nevertheless, it is still elusive about a comprehensive understanding of its precise functional roles and underlying molecular mechanisms during reproduction of neo-tetraploid rice. Here, we demonstrated that *OsNRFG6* executed a constitutive expression pattern and encoded proteins localizing in perinucleus and endoplasmic reticulum. Subsequently, four independent mutant lines of *OsNRFG6* within neo-tetraploid rice background were further identified, all displaying low seed-setting rate due to abortive embryo sacs and defective double fertilization. RNA-seq and RT-qPCR revealed a significant down-regulation of *OsNRFG6* and female reproductive genes such as *OsMEL1* and *LOG* in ovaries prior to and post-fertilization, attributing this effect to *OsNRFG6* mutation. Furthermore, through yeast-two hybrids, bimolecular fluorescence complementation assays, and luciferase complementation imaging assays, it was determined that OsNRFG6 could interact with itself and two female reproductive proteins (LOG and OsDES1) to form protein complexes. These results elucidate the reproductive functions and molecular pathway governed by *OsNRFG6* in regulating fertility of neo-tetraploid rice, offering insights into molecular understanding of fertility improvement in polyploid rice.

**Supplementary Information:**

The online version contains supplementary material available at 10.1186/s12284-024-00720-0.

## Introduction

Polyploidy show robust growth characterized by stronger biosynthesis, increased nutrient composition, and heightened adaptability to stress and plant evolution (Corneillie et al. 2019; Yu et al. 2021a; Wang et al. 2022). Tetraploid hybrid rice holds substantial promise for yield increase via superposing polyploidy advantage and heterosis (Chen et al. 2019; Ghaleb et al. 2020). But its breeding process was limited by reproductive dysfunction associated with polyploidy, including pollen sterility, embryo sac sterility, delayed fertilization, irregular embryogenesis, as well as abnormal endosperm development (Wu et al. 2015; Li et al. 2017, 2023). Relative to extensive studies in polyploidy pollen sterility, it is more difficult in cytological observation and genetic analysis about embryo sac or embryo, resulting in limited understanding about the regulation of embryo sac development and embryogenesis in autotetraploid rice (ATR).

Chinese scientists had successfully bred fertile tetraploid rice, including Polyploid Meiosis Stability (PMeS) rice and neo-tetraploid rice (NTR) (He et al. 2011; Guo et al. 2017; Ghaleb et al. 2020; Liu et al. 2023). PMeS lines, with stable meiotic behaviors and high seed setting rete, were bred from progenies of tetraploid intersubspecific hybrid rice, HT99104 (*japonica*) × Shuhui362 (*indica*) (He et al. 2011). NTR lines are new tetraploid rice germplasms developed from the crossing and directional selection of ATR lines (Jackson-4x and 96025-4x) by our group, which had the ability to overcome the polyploidization sterility when they crossed with typical ATR lines with low fertility (Guo et al. 2017; Ghaleb et al. 2020; Yu et al. 2020). These fertile tetraploid rice germplasms have provided opportunities for identifying genes related to autotetraploid sterility. Our previous studies have evaluated 15 NTR lines to assess their yield traits, reproduction and gene expression (Chen et al. 2019; Ghaleb et al. 2020; Yu et al. 2020). Relative to diploid and NTR lines, substantial differences were found in expression levels of genes, miRNA as well as long non-coding RNA during embryo sac development of ATR, such as meiotic genes (Li et al. 2016, 2017; Guo et al. 2017; Ku et al. 2022).

The origin of regulation for high fertility of NTR or PMeS lines might relate to those improved genotypes of key genes formed during germplasm evolution (Koide et al. 2020). For example, the evolved autotetraploid *Arabidopsis arenosa* (fertile) has formed alternate alleles of important fertility genes like *ASY1*, *ASY2*, *ACA8*, and *AGC1.5* to overcome its unstable meiotic chromosome axes, aberrant crossover interference, and defective pollen tube tip growth (Morgan et al. 2020, 2021; Westermann et al. 2024). Similarly, 222–324 genes with different alleles between 13 fertile NTR lines and two sterile ATR parental lines (Jackson-4x and 96025-4x) were identified in genomic comparison (Bei et al. 2019; Yu et al. 2020, 2021b). The NTR alleles of these genes were marked as non-parental alleles, which might be formed because of accumulated natural mutations during material breeding process. Furthermore, our previous studies had constructed mutants of 11 genes with non-parental alleles by CRISPR/Cas9 technology, including *LOC_Os06g40030* encoding lectin receptor-like protein kinase (named as NEO-TETRAPLOID RICE FERTILITY RELATED GENE 6, OsNRFG6). The *Osnrfg6* mutants in NTR line Huaduo 1 (H1) background exhibited low seed-setting rate and normal pollen fertility, suggesting that *OsNRFG6* might affect embryo sac development or double fertilization of NTR (Yu et al. 2020).

Given the potential importance of *OsNRFG6* in the high fertility of NTR, further studies are warranted to elucidate its roles. In this study, we identified four mutant lines of *OsNRFG6* for phenotypic characterization and cytological observation, and further elucidated the expression pattern, gene regulation and protein interaction involving *OsNRFG6*. These findings are anticipated to shed light on the role of *OsNRFG6* during female reproduction in tetraploid rice.

## Results

### Phylogenetic and Expression Pattern Analyses of *OsNRFG6*

Lectin receptor-like kinases (LecRLKs) in rice were classified as C-type (one member), L-type (72 members) and G-type (100 members) based on their diversities in lectins and kinase domains (Vaid et al. 2012). The coding sequence (CDS) of *OsNRFG6* consists of 2442 nucleotides, encoding an 813-amino acid protein, which is a putative G-type LecRLK consisting of an extracellular signal peptide (SP) domain, a classic B-lectin domain (36–144 aa), an S_locus_glycop (SLG, 248–308 aa) domain, a PAN domain (325–407 aa), a transmembrane (TM, 459–481 aa) domain, and an intracellular serine/threonine kinase (STK, 524–803 aa) domain (Fig. S1). Furthermore, 16 OsNRFG6 orthologs (Identity > 67%) were identified among different grass species, which had high conserved kinase domain. OsNRFG6 was classified into a clade together with monocot species like *Aegilops tauschii subsp*, *Triticum aestivum*, *Hordeum vulgare*, *Lolium perenne*, *Lolium rigidum* and *Brachypodium distachyon* (Fig. S1, S2).

**Fig. 1:**
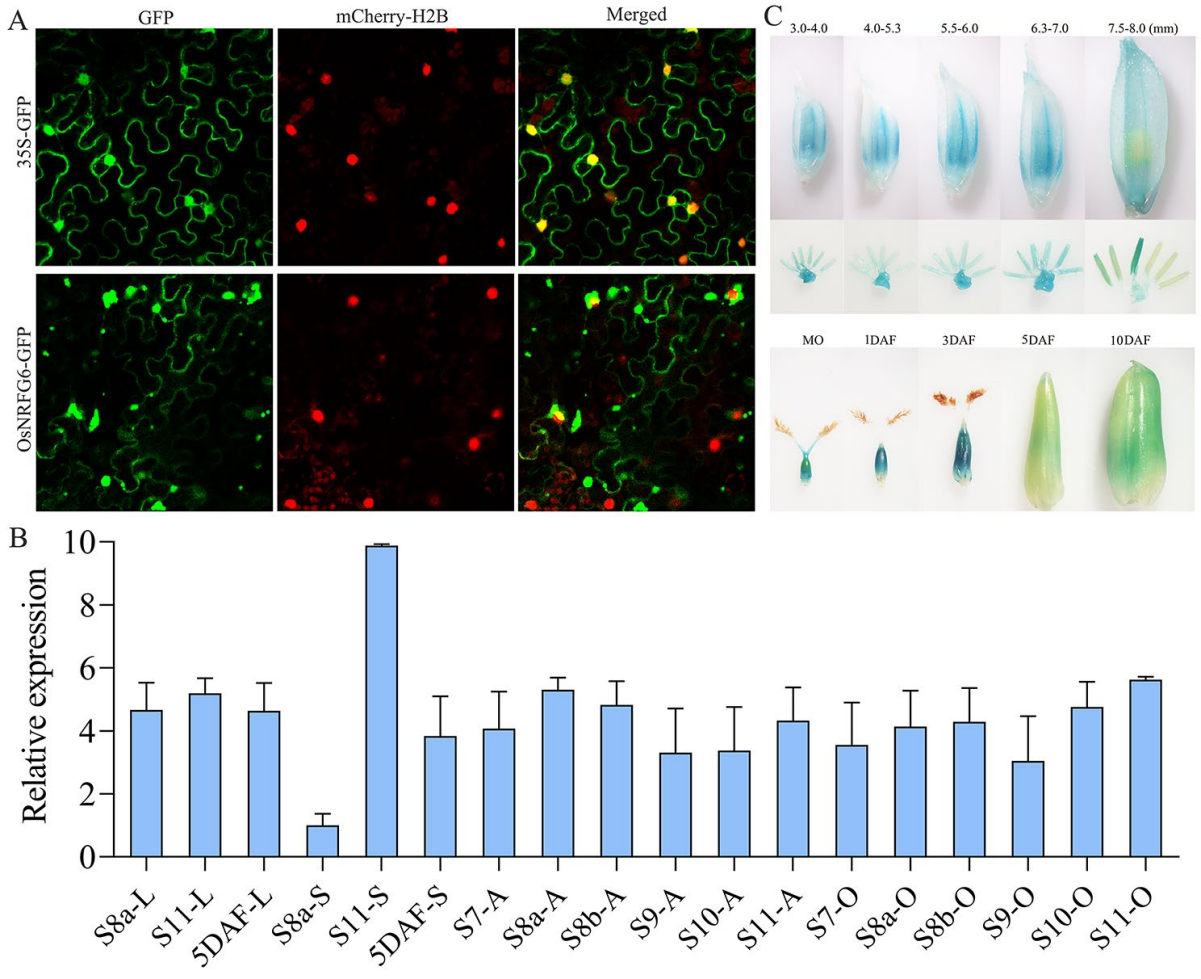
Subcellular localization of OsNRFG6 and expression pattern of *OsNRFG6* in rice. (**A**) Subcellular localization of OsNRFG6-GFP fusion protein in *Nicotiana benthamiana* leaf epidermal cells. H2B-mCherry indicates the nuclear localization marker. 35 S-GFP was used as the control. (**B**) Relative expression of *OsNRFG6* in various tissues. L, leaf; S, sheath; A, anthers; O, ovaries; 5DAF, 5 days after flowering; S7 to S11, stages of anther development. (**C**) GUS staining in various tissues of pro*OsNRFG6*-GUS transgenic plants of developing spikelets and pistils. The lengths of observed spikelets or developmental stages of ovaries were listed on the top side. MO, mature ovary. DAF, days after flowering. The error bars indicate the SD with *n* = 3

To character the expression pattern of *OsNRFG6*, subcellular localization, public gene expression databases (RiceXPro and Rice eFP Browser), reverse transcription quantitative real-time PCR (RT-qPCR), and promoter-GUS staining assay were performed. Different with free GFP signals, most of OsNRFG6-GFP couldn’t be co-localized with H2B-mChreey signal (Fig. 1A). The fluorescent signals of OsNRFG6-GFP formed spotted fluorescence around nuclei of *Nicotiana benthamiana* leaves, but only a handful of OsNRFG6-GFP signals could localize in nuclei (Fig. 1A, Fig. S3A). Excepted the spotted fluorescent signals, OsNRFG6-GFP signals mainly localized on the endoplasmic reticulum, which could well co-localized with mCherry-HDEL (Fig. S3B). In RiceXPro database, *OsNRFG6* highly expressed in leaves, leaf sheath, roots, stems, inflorescence, pistils, lemma, palea, ovaries, embryo, and endosperms at various developmental stages, but low in developing anthers (Fig. S4A). In Rice eFP Browser database, *OsNRFG6* mainly expressed in developing inflorescence, seeds, and leaves (Fig. S4B). Similarly, *OsNRFG6* constitutively expressed in leaves, stems, anthers and ovaries of wild type neo-tetraploid rice (NTR) line Huaduo1 (H1) at different developmental stages in RT-qPCR assay (Fig. 1B), which indicated *OsNRFG6* may play an important role in regulating reproductive development in H1. In GUS reporter system derived by *OsNRFG6* promoter, GUS signals could be detected at different developmental stages in florets, especially in the anthers and ovaries (Fig. 1C). The GUS signals were observed in the ovule wall and the cavity of ovule by the semi-thin sections (Fig. S4C). Taken together, *OsNRFG6* functioned under a constitutive expression pattern.

### *Osnrfg6* Mutants Displayed Low Seed Setting Rate

To further examine the reproductive roles of *OsNRFG6* in NTR, our group knocked out the *OsNRFG6* gene under the H1 background by CRISPR/Cas9 technology in our previous study (Yu et al. 2020). Four homozygous mutants (designated as *Osnrfg6-1* to *Osnrfg6-4*) were identified by sanger sequencing analysis from T_3_ generation *Osnrfg6* mutants (Fig. S5A). When predicting the protein sequence of OsNRFG6 in *Osnrfg6* based on their mutant genotypes, frameshift translation and premature translation termination were found in *Osnrfg6-2* and *Osnrfg6-4*, while 14 and 1 amino acids of the OsNRFG6 were deleted in *Osnrfg6-1 and Osnrfg6-3*, respectively (Fig. S5B).

**Fig. 2:**
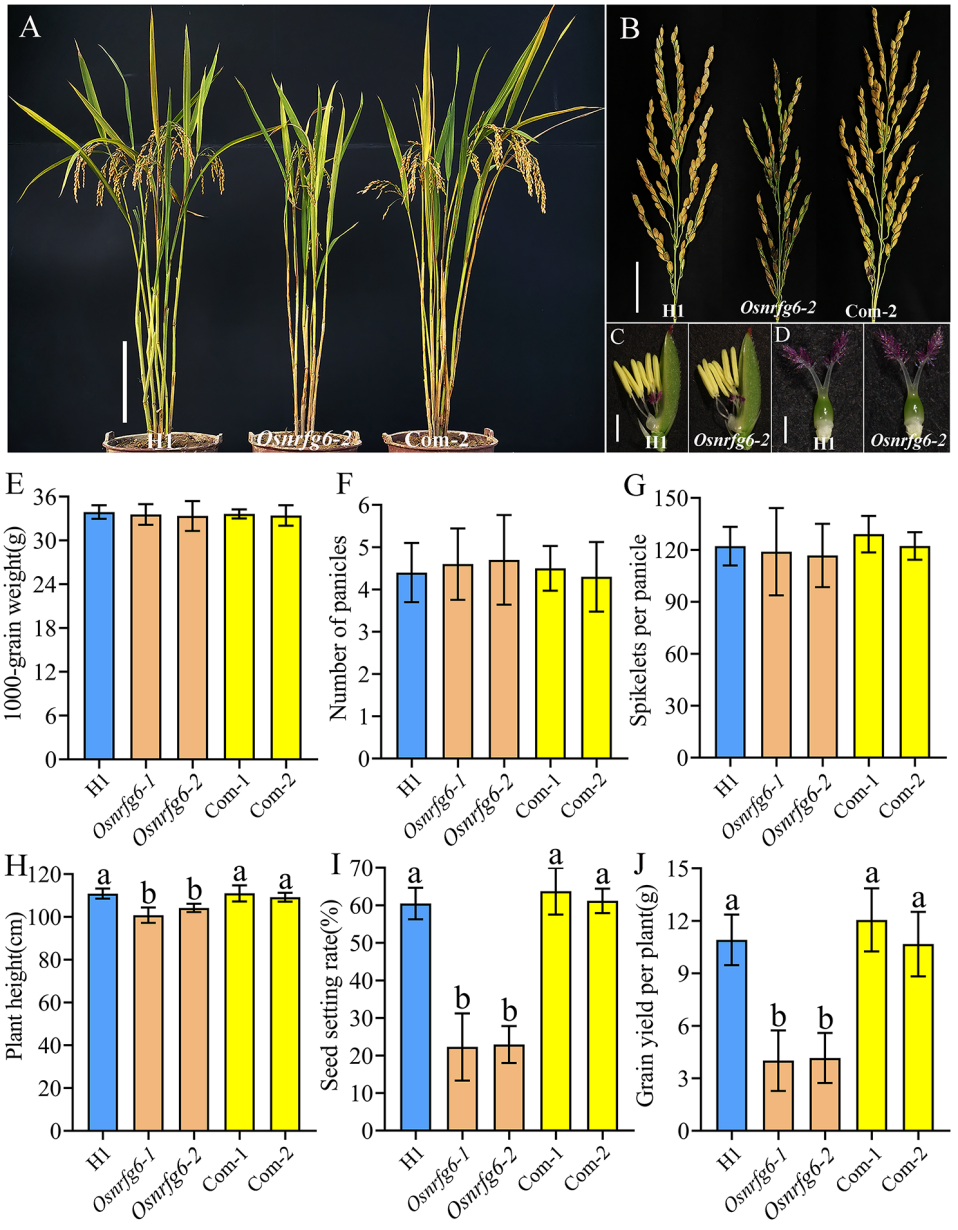
Phenotypic characterization of *Osnrfg6*. (**A**) Plants morphology, (**B**) mature panicles, (**C**) mature anthers, (**D**) mature pistils, (**E**) 1000-grain weight, (**F**) number of panicles, (**G**) spikletes per panicle, (**H**) plant height, (**I**) seed-setting rate, and (**J**) grain yield per plant in H1, *Osnrfg6* and complementary plants, respectively. Scale bar = 20 cm (**A**), 4 cm (**B**), and 1 mm (**C**-**D**). Error bars indicate the SD with *n* = 10. Different letters indicate significant differences (*P* < 0.01, Least significant difference)

Compared with H1, *Osnrfg6* exhibited normal vegetative growth with no clear differences in flower organ morphology, 1000-grain weight, number of panicles and spikelets per panicle (Fig. 2A-G; Fig. S6A-I). But the plant height of *Osnrfg6* mutants were shorter than H1 (Fig. 2H; Fig. S6J). Moreover, the seed-setting rates of *Osnrfg6-1* (22.27%) and *Osnrfg6-2* (22.93%) were significantly lower than H1 (60.46%) (Fig. 2I), leading to significant reduction of grain yield per plant in *Osnrfg6-1* (4.01 g) and *Osnrfg6-2* (4.16 g) (Fig. 2J). Meanwhile, two complementation transgenic lines named Com-1 and Com-2 were constructed by transforming *Osnrfg6-2* with a 4503 bp *OsNRFG6* genomic DNA fragment to verify the relationship between *Osnrfg6* mutation and its infertility. The seed-setting rate of Com-1 (63.74%) and Com-2 (61.20%) were similar to that of H1 and significantly higher than *Osnrfg6-2* (Fig. 2I). Similarly, the defect of *Osnrfg6* in plant height and grain yield per plant were rescued both in Com-1 and Com-2 (Fig. 2H, J). These results indicated that *OsNRFG6* plays important roles in regulating seed-setting rate of NTR lines.

### *OsNFRG6* did not Affect Pollen Germination and Pollen Tube Growth

**Fig. 3:**
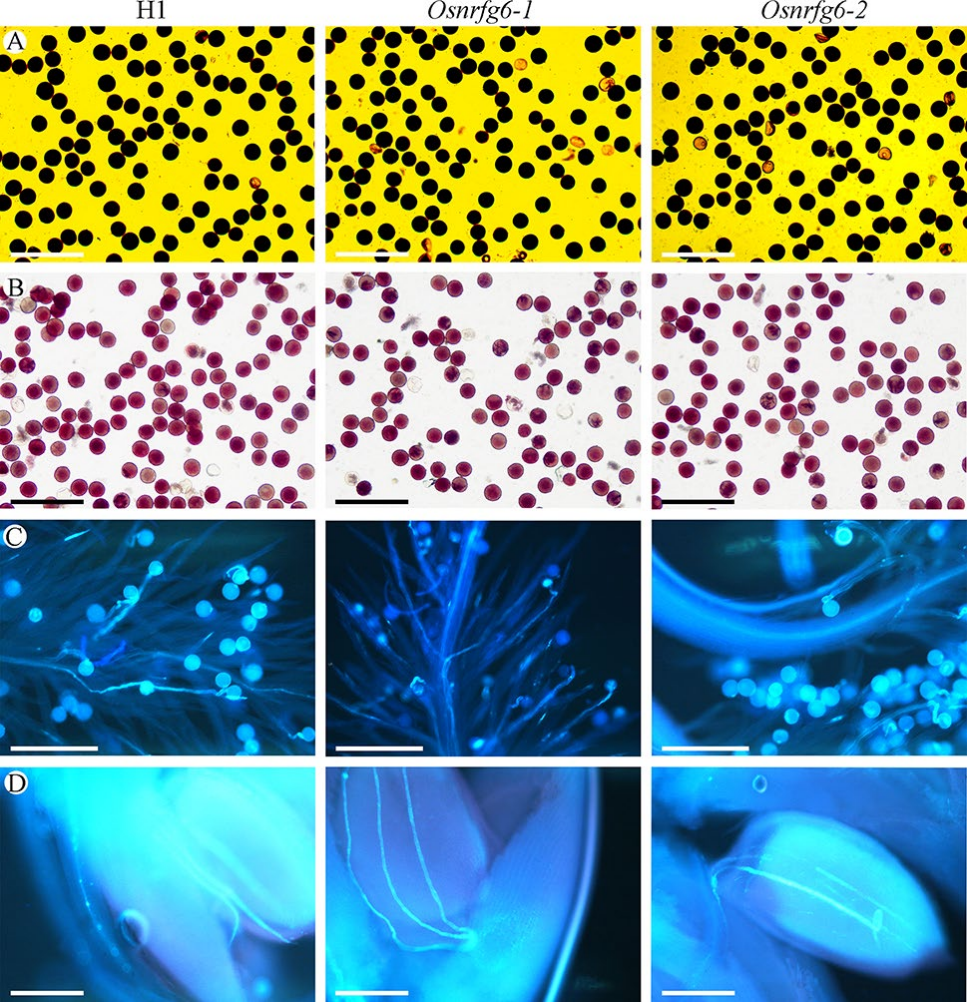
Observation of pollen grains and pollen tubes in H1 and *Osnrfg6*. (**A**) I_2_-KI staining pollen grains, (**B**) 1% TTC staining pollen grains, (**C**) in vitro pollen grains germination, and (**D**) in vitro pollen tubes elongation of H1, *Osnrfg6-1* and *Osnrfg6-2*. The observed samples were stained with aniline blue in (**C**) and (**D**). Bars = 200 μm

Semi-thin sectioning showed that H1, *Osnrfg6-1* and *Osnrfg6-2* had no obvious differences in the structure of mature anthers (Fig. S7A). Moreover, five staining methods were used to examine the pollen fertility of *Osnrfg6*, including I_2_-KI for calculation of abortive pollen, eosin B for whole mount observation, 1% TTC for pollen viability, optical brightener for pollen intine and auramine O for pollen exine. Similar to H1 (96.73%), 90.15% and 92.00% pollen grains normally developed in *Osnrfg6-1* and *Osnrfg6-2* (Fig. 3A, Fig. S7B). Regardless of *OsNFRG6* genotypes, the intravital TTC-stained pollen grains turned to deep red (high vitality) (Fig. 3B), the pollen grains stained by optical brightener turned to blue (normal intine) (Fig. S7C), and the pollen grains stained by auramine O turned to blight green (intact exine) (Fig. S7D). At 30 min after flowering, normal pollen grains germinations were observed on the stigma of H1 (95.82%), *Osnrfg6-1* (94.59%) and *Osnrfg6-2* (90.28%) (Fig. 3C). At 2 h after flowering, the germinated pollen tubes arrived the micropyles of H1 (98.48%), *Osnrfg6-1* (97.53%) and *Osnrfg6-2* (96.30%) (Fig. 3D). These results indicated that the pollen development, pollen germination and pollen tubes growth were normal in *Osnrfg6* mutants.

### *OsNRFG6* was Required for Embryo Sac Development of Neo-Tetraploid Rice

**Fig. 4:**
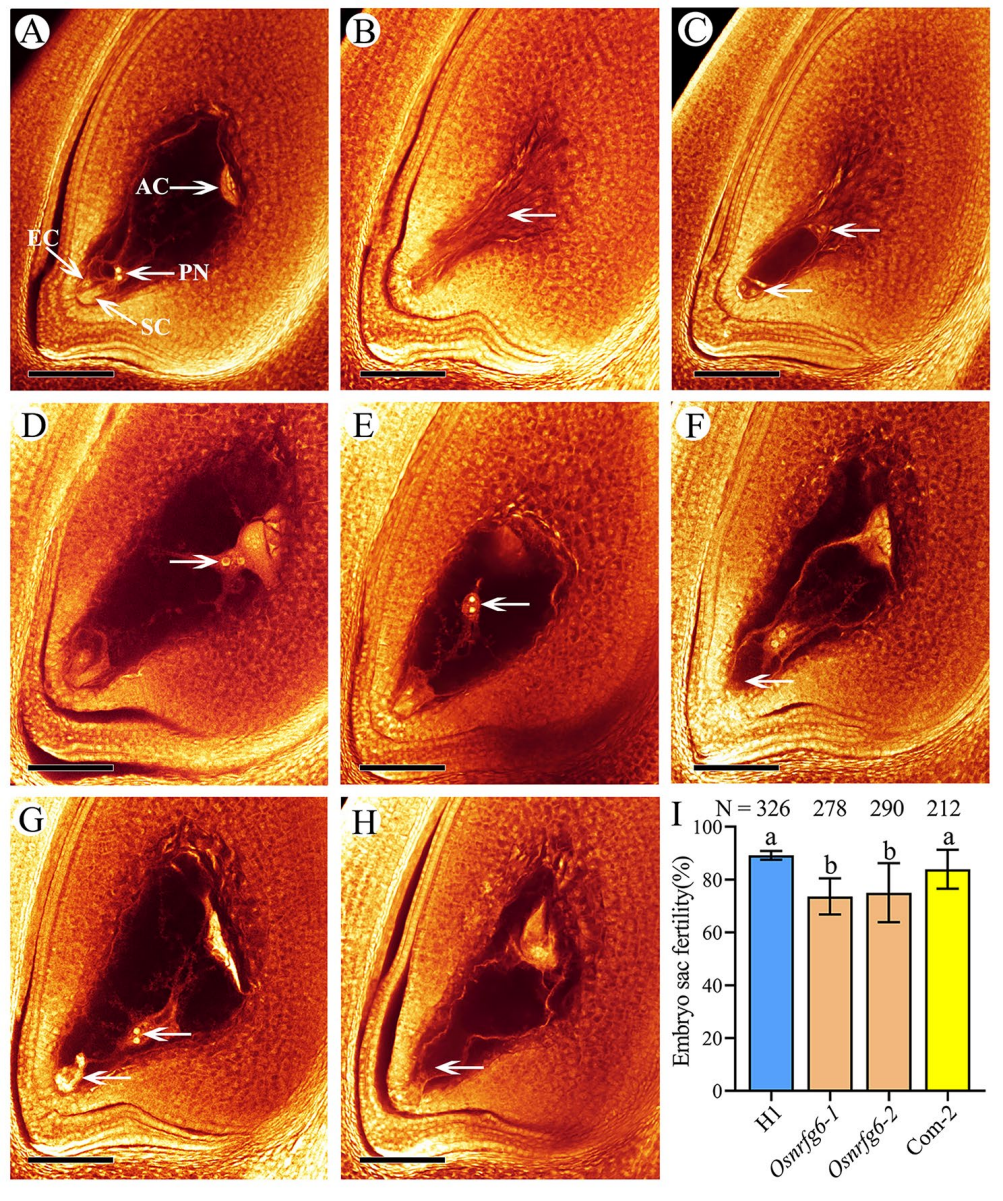
WE-CLSM observation of mature embryo sacs in H1 and *Osnrfg6*. (**A**) Normal embryo sac in H1. (B-H) Main abnormalities of embryo sacs in *Osnrfg6*. (**B**) Degenerated embryo sac without the differentiation of embryo sac cavity (arrow). (**C**) Arrested developmental embryo sac (arrows). (**D**-**E**) Atypical positioning polar nuclei (arrows) in *Osnrfg6*. (**F**) Embryo sac without egg apparatus (arrow) (**G**) Atypical positioning polar nuclei and egg apparatus degeneration (arrows). (**H**) Degeneration female germ unit (arrow). (**I**) Embryo sac fertility of H1, *Osnrfg6* and complementary plants. AC, antipodal cell; EC, egg cell; PN, polar nucleus; SC, synergid cell. Scale bars = 100 μm. Error bars indicate the SD. N represents the number of observed embryo sacs. Different letters indicate significant differences (*P* < 0.01, Least significant difference)

The mature embryo sac fertility in both H1 and *Osnrfg6* was assessed by using the whole-mount eosin B-staining confocal laser scanning microscopy (WE-CLSM). The embryo sac structure in H1 is similar to that of diploid rice, consisting of one central cell flanked by two polar nuclei, one egg cell, two synergids and three antipodal cells (Fig. 4A, Video S1). During its developmental process, the megaspore mother cell (Fig. S8A) underwent meiosis to form a tetrad (Fig. S8B); Subsequently, three megaspore mother cells located near the micropylar end gradually degenerated, while the remaining one located near chalazal end enlarged to form a functional megaspore (Fig. S8C-D). This megaspore underwent three times rounds of mitotic division, resulting in the formation of mono-nuclear (Fig. S8E), two-nucleate (Fig. S8F), four-nucleate (Fig. S8G), and eight-nucleate embryo sac (Fig. S8H). Ultimately, one chalazal end nucleus and one micropylar end nucleus moved, and fused to form polar nuclei at micropylar end. Other chalazal end nuclei became antipodal cells, while other micropylar end nuclei formed an egg apparatus, comprising of an egg cell and two synergids (Fig. S8I).

The mature embryo sac fertility of *Osnrfg6-1* (73.62%) and *Osnrfg6-2* (75.04%) is significantly lower than H1 (93.67%) (Fig. 4I, Table S1). The abnormalities of embryo sac in *Osnrfg6* were involved in degeneration (5.86–8.27%) (Fig. 4B), arrested development (0.00-4.32%) (Fig. 4C), abnormal position of polar nuclei (4.83–6.47%) (Fig. 4D-E, Video S2), egg apparatus degeneration (4.14–4.68%) (Fig. 4F, Video S3), abnormal polar nuclei and egg apparatus (0.36–4.14%) (Fig. 4G), and female germ unit degeneration (3.24–4.48%) (Fig. 4H, Video S4). The reduced embryo sac fertility of *Osnrfg6-2* was rescued in Com-2 (83.91%) (Fig. 4I).

### *OsNRFG6* was Required for Normal Fertilization of Neo-Tetraploid Rice

**Fig. 5:**
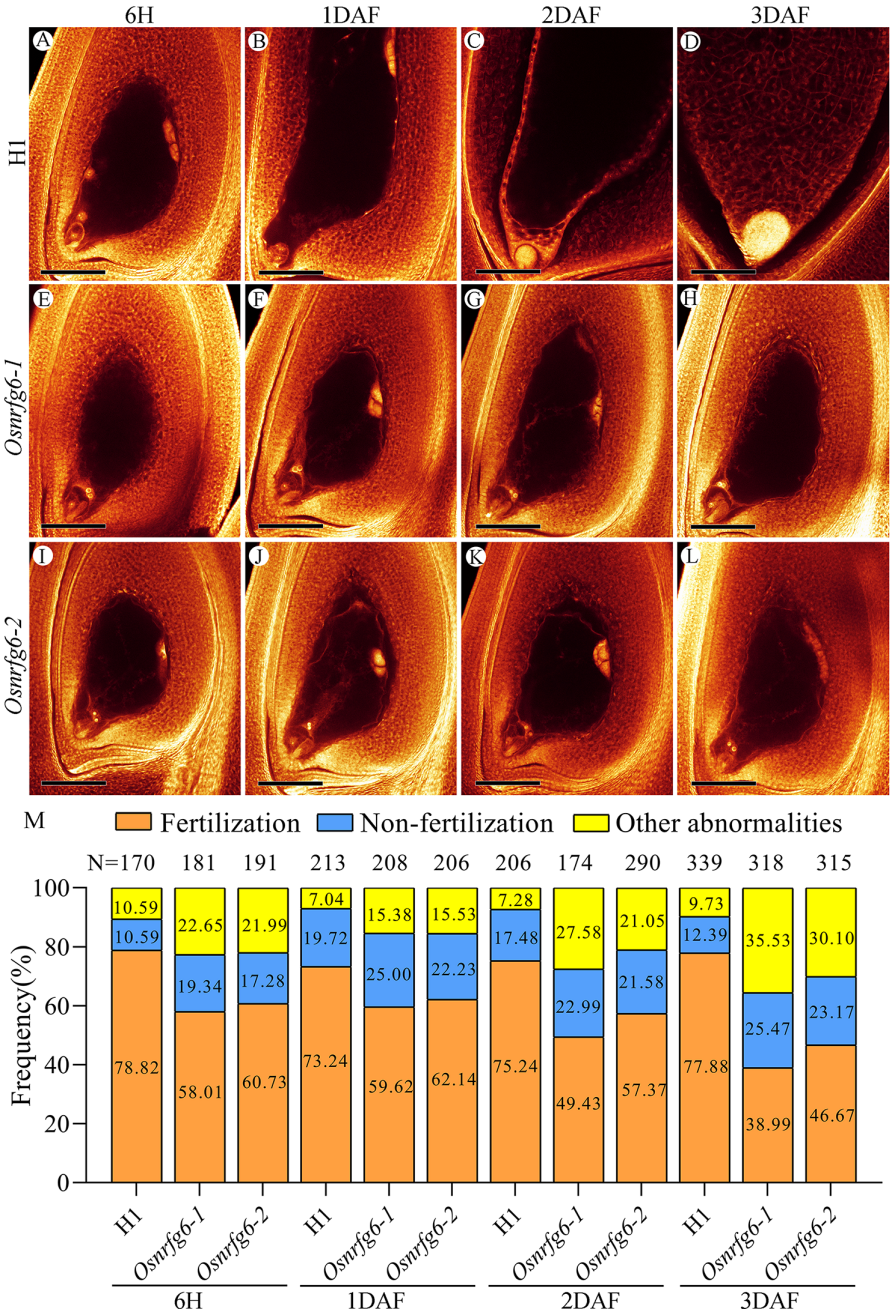
WE-CLSM observation of double fertilization development and embryogenesis in H1 and *Osnrfg6*. (**A**-**D**) Normal double fertilization development and embryogenesis in H1. (**E**-**L**) Abnormal double fertilization development and embryogenesis in *Osnrfg6*. (**M**) Data statistics of double fertilization development and embryogenesis in H1 and *Osnrfg6*. N represents the number of observed embryo sacs. Scale bars = 150 μm. 6 H, 6 h after flowering; DAF, day after flowering

Additionally, WE-CLSM was also employed to observe the characteristics of embryogenesis at 6 h after flowering, 1 day after-flowering (1DAF), 2DAF, and 3DAF in H1 and *Osnrfg6*. At 6 h after flowering, the primary endosperm nucleus in 78.82% H1 samples had initiated nuclear division to form multiple endosperm nuclei, while only 58.01–60.73% *Osnrfg6* samples exhibited normal nuclear division (Fig. 5A, M). In this stage, 17.28–19.34% *Osnrfg6* samples were unfertilized (Fig. 5E, I), and 21.99–22.65% *Osnrfg6* samples were in other abnormalities, including embryo sacs degeneration, arrested development embryo sacs, abnormal position of polar nuclei embryo sacs and other abnormal embryo sacs (Fig. S9 A-C, H-J).

From 1DAF to 3DAF, the ovaries continually enlarged, the zygotes differentiated into spherical or pear shaped embryoids, and those endosperm nuclei around the central cell increased and turned into endosperm cells in 73.24–77.88% H1 samples (Fig. 5B). By contrast, abnormal ovaries continually increased in *Osnfrg6* during this period. 21.58–25.47% *Osnfrg6* samples were unfertilized, which always kept the size and a structure as same as normal mature embryo sac (Fig. 5F-H, J-L). As developmental process went on, more and more various abnormalities were observed in *Osnfrg6*, which increased from 15.38 to 35.53%, involving in ovaries with degraded embryo sacs, arrested development embryo sacs, unfertilized ovules with dislocated polar nuclei, and unfertilized ovules with abnormal hyperplasia in nuclear cells (Fig. S9D-G, K-N, Fig. S10). These results indicated that *OsNRFG6* also plays important roles in maintaining normal fertilization and embryogenesis of NTR lines.

### Mutations in *OsNRFG6* Altered Expression Profile of key Genes for Reproduction

**Fig. 6:**
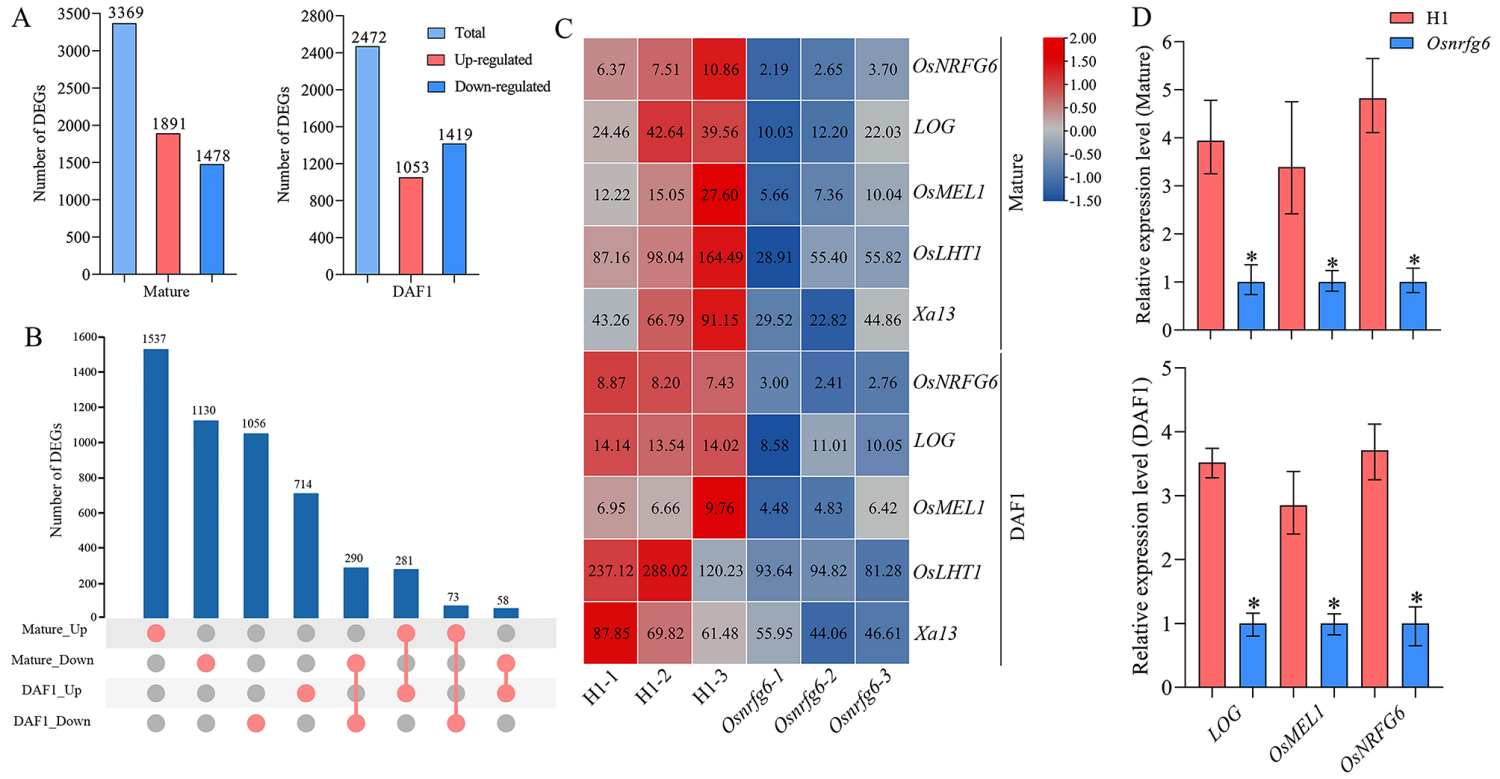
Reproductive genes differentially expressed during development of embryo sac and double fertilization in *Osnrfg6*. (**A**) Differentially expressed genes (DEGs) analysis in the ovaries of H1 and *Osnrfg6* at mature embryo sac stage and 1 day after flowering stage. (**B**) Venn analyses of DEGs in the ovaries at two stages. (**C**-**D**) Expression levels of key down-regulated genes related to seed-setting rate in RNA-seq (**C**) and RT-qPCR (**D**) analyses of H1 and *Osnrfg6*. Error bars indicate the SD with *n* = 3. Asterisks indicate significant differences (*P* < 0.05, Student’s test)

To identify the putative and related genes regulated by *OsNRFG6*, we performed RNA-seq analysis of the ovaries in H1 and *Osnrfg6* at mature embryo sac stage and 1 day after flowering stage, respectively. More than 6.17 Gb of clean data were yielded from each sample. Clean data from each sample were mapped to the Nipponbare (*Oryza sativa* ssp. *japonica*) reference genome using HISAT2. The clean reads of each sample were sequenced with the reference genome, and the alignment efficiency ranged from 94.77 to 95.62%. Compared with H1, 3369 significant differentially expressed genes (DEGs) were detected in mature embryo sac of *Osnrfg6*, including 1891 up- and 1478 down-regulated genes (Fig. 6A). Meanwhile, 2472 DEGs were identified in 1DAF ovaries of *Osnrfg6*, involved in 1053 up- and 1419 down-regulated genes (Fig. 6A). The Kyoto Encyclopedia of Genes and Genomes (KEGG) pathway analysis revealed that two group DEGs were together enriched in plant-pathogen interaction, plant hormone signal transduction and MAPK signaling pathway-plant (Fig. S11).

Venn analysis revealed that 281 up- and 290 down-regulated DEGs shared in two stage samples, which were designed as coDEGs (Fig. 6B; Table S2, S3). Expression of *OsNRFG6* was significantly reduced during ovary development in *Osnrfg6*. In addition, five coDEGs with function in rice reproduction attracted our attention, including *LOG* (*LOC_Os01g40630*), *OsMEL1* (*LOC_Os03g58600*), *OsLHT1* (*LOC_Os08g03350*), and *Xa13* (*LOC_Os08g42350*). *OsMEL1* and *LOG* both significantly down-regulated at two stages (Fig. 6C). *OsMEL1* regulated meiosis and its loss-of-function mutants resulted in embryo sac degeneration (Nonomura et al. 2007). *LOG* encoded a cytokine-activating enzyme which affected female fertility by regulating the pistil and ovule development (Kurakawa et al. 2007). Then RT-qPCR analysis further verified that mutation of *OsNRFG6* caused significant reduction in expression of *OsNRFG6*, *OsMEL1* and *LOG*, which were consistent with the RNA-seq results (Fig. 6D). These results suggested that *OsNRFG6* may regulate embryo sac development and double fertilization by affecting the expression profiles of key reproductive genes.

### OsNRFG6 Formed Homodimers and Protein Complexes with LOG and OsDES1

**Fig. 7:**
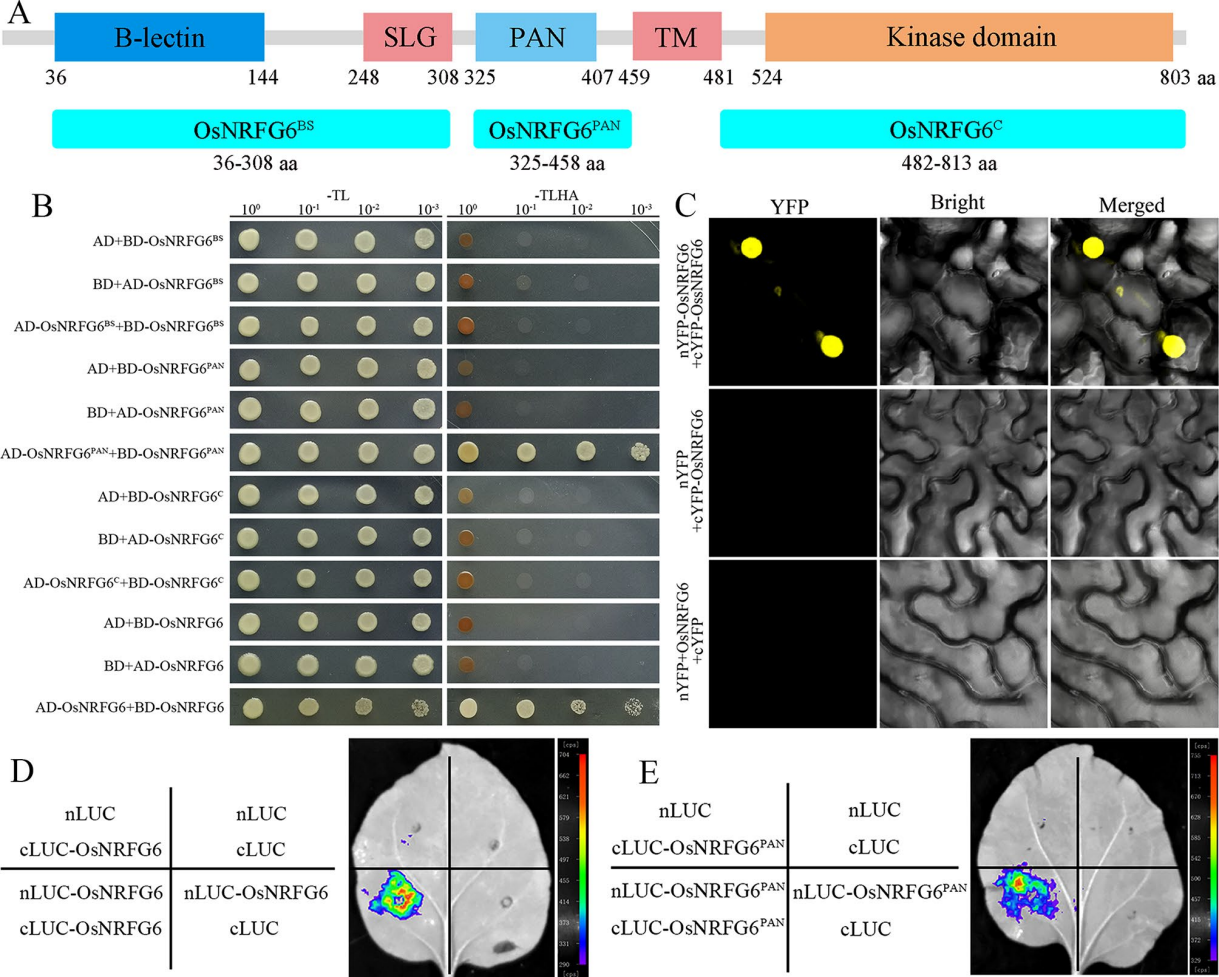
The protein properties of the OsNRFG6 protein. (**A**) Schematic diagram of the OsNRFG6 protein. (**B**) The yeast-2-hybrid assay between truncated OsNRFG6 proteins and its full-length protein. “-TL” and “-TLHA” indicate SD/-Trp-Leu and SD/-Trp-Leu-His-Ade medium. (**C**-**E**) OsNRFG6 interacted with itself via its PAN domain in *Nicotiana benthamiana* leaf cells, as seen in BiFC and LCI assays. SLG, S-locus glycoprotein domain; TM, transmembrane domain

To reveal the protein interaction of OsNRFG6, we then performed yeast two-hybrid (Y2H) assay, luciferase complementation imaging assay (LCI), and bimolecular fluorescence complementation assay (BiFC). Excepted full length OsNRFG6 protein, OsNRFG6 were splitted into three fragments for follow research: OsNRFG6^BS^ with B-lectin domain and SLG domain (36–308 aa), OsNRFG6^PAN^ with PAN domain (325–458 aa), and OsNRFG6^C^ with a kinase domain (482–813 aa) (Fig. 7A). Y2H exhibited that OsNRFG6 has the ability to form homodimers via its PAN domain rather than OsNRFG6^BS^ or OsNRFG6^C^ fragments (Fig. 7B), which were further verified by LCI and BiFC assays (Fig. 7C-E, S12).

**Fig. 8:**
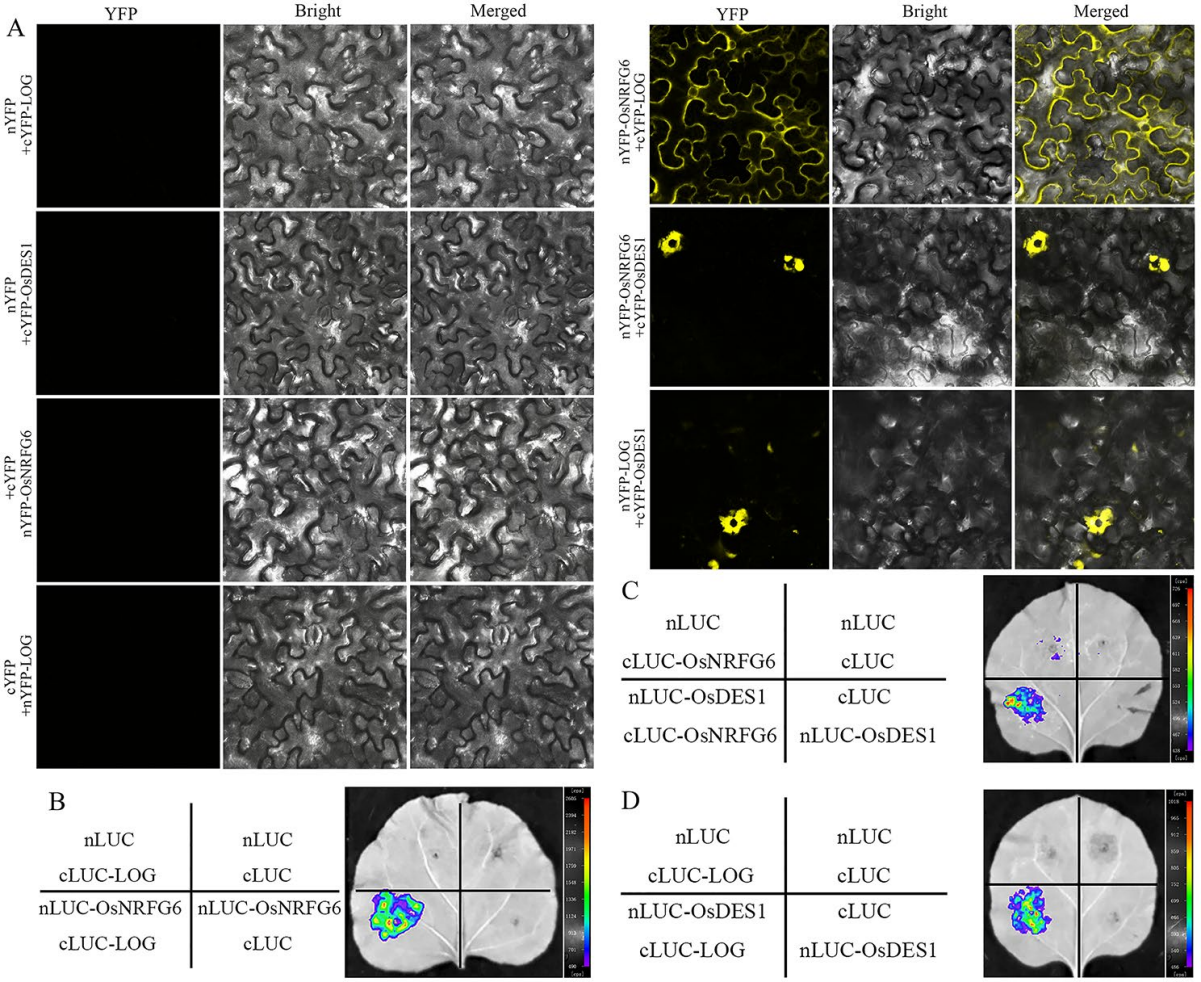
OsNRFG6 physically interacted with LOG and OsDES1. (**A**) The BiFC tests, and (**B**-**D**) LCI experiments revealed the protein interactions among OsNRFG6, LOG and OsDES1 in *Nicotiana benthamiana* leaf epidermis cells

Furthermore, two important female reproductive proteins, LOG and OsDES1, showed the ability to physically interacted with OsNRFG6 both in BiFC and LCI assays (Fig. 8A-C). OsDES1 interacted with LOG to regulate embryo sac development and fertilization (Hu et al. 2023), whose mutant caused similar phenotypic defects with *Osnrfg6*. Similarly, the protein interaction between OsDES1 and LOG were verified in our BiFC and LCI assays (Fig. 8A-C). Taken together, OsNRFG6, OsDES1 and LOG might form protein complexes required for embryo sac development and fertilization of NTR.

## Discussion

Polyploid rice breeding was limited by its reproductive dysfunction, including abortive pollen grains, defective embryo sac as well as abnormal fertilization. For example, 02428-4x and Taichung 65-4x had only 55.48 ~ 62.15% embryo sac fertility, involving in embryo sac degeneration, and abnormal position and number of polar nuclei (Li et al. 2017, 2020). In addition, the fertilization, embryo and endosperm development were abnormal in autotetraploid rice (ATR) lines like 02428-4x with 68.84% abnormal fertilization rate, involving in unsuccessful fertilization, single-fertilization and enlarged ovary without double-fertilization (Li et al. 2020). The abnormalities of embryo sacs and double fertilization were overcome in neo-tetraploid rice (NTR), such as five reported NTR lines (H1, H3, H4, H5, and H21) with 76.04 ~ 95.47% embryo sac fertility (Bei et al. 2019; Chen et al. 2019; Li et al. 2023). In this study, *Osnrfg6* mutant exhibited the embryo sac degeneration and abnormal polar nuclei similar to infertile ATR lines (Fig. 4). Moreover, *Osnrfg6* also shared similar abnormalities during double-fertilization process with infertile ATR lines, including unfertilized, single-fertilized, and inflated ovules (Fig. 5, S9, S10), but not in NTR lines (Li et al. 2023). During the breeding process of NTR lines, *OsNRFG6* had formed an improved genotype different from the parental lines (Yu et al. 2020). Similar mechanism has been found that improved genotypes of fertility genes helped autotetraploid *Arabidopsis arenosa* to adjust its defective meiosis process and pollen tube tip growth (Morgan et al. 2020, 2021; Westermann et al. 2024). Thus, the non-parental allele of *OsNRFG6* may play important roles in the formation of high fertility of NTR lines.

*OsNRFG6* encodes a G-type lectin receptor-like protein kinase (LecRLK), containing lectin domain, transmembrane domain and intracellular protein kinase domain, which belongs the receptor-like kinases (RLKs) family. RLKs have been reported to play important roles in reproductive process of rice, including *OsMSP1*, *OsLecRK-S.7*, and *MIL2* regulating microsporocyte number and anther development (Nonomura et al. 2003; Zhao et al. 2008; Hong et al. 2012; Peng et al. 2020); *OsMSP1*, *OsDEES1*, and *MIL2* regulating megasporocyte number and embryo sac development (Nonomura et al. 2003; Zhao et al. 2008; Hong et al. 2012; Wang et al. 2012); RUPO-OsHAK1/19/20 and OsDAF1-OsINP1 molecular modules regulating pollen tubes growth (Liu et al. 2016; Zhang et al. 2020). In this study, we verified the reproductive functions of an uncharacterized lectin receptor-like protein kinase gene *OsNRFG6* in NTR by CRISPR/Cas9 technology and genetic complement experiment. *OsNRFG6* mainly effects embryo sac development and fertilization of NTR lines, but not in the pollen development.

In the ovaries with mature embryo sac or developing embryo, loss function of *OsNRFG6* caused significant downregulation of fertility related genes, such as *Xa13*, *LOG*, *OsMEL1*, and *OsLHT1*, which might relate to its defective female reproduction (Fig. 6). Mutation of *OsMEL1* would cause arrested development of female gametophytes at meiotic stages, leading to embryo sac abortion (Nonomura et al. 2007), while mutation of *LOG* caused abnormal pistil development and female sterility (Kurakawa et al. 2007). In addition, our previous studies found that *OsMEL1* differentially expressed during reproduction in comparative analyses related to ATR lines, including meiotic anthers between 02428-2x/02428-4x (Li et al. 2018), meiotic anthers between NTR line H1 and ATR line T44 (Wu et al. 2020), and developing ovaries between NTR lines (Huaduo3 and Huaduo8) and ATR lines (Huajingxian74-4x and Huanghuazhan-4x) (Guo et al. 2017; Ghaleb et al. 2020). These results suggested a candidate relationship among *OsNRFG6* and key female reproductive genes in regulating embryo sac development of ATR.

Moreover, we found that OsNRFG6 has the ability to form homodimers via its PAN domain rather than OsNRFG6^BS^ or OsNRFG6^C^ fragments, which were verified by Y2H, LCI and BiFC assays (Fig. 7, S12). In previous studies, similar mechanism of lectin receptor-like kinases forming homodimers via PAN domain has been reported, such as OsLecRK5 (Wang et al. 2020), and PWL1 (Xu et al. 2023). We further identified the protein interactions among OsNRFG6-LOG, OsNRFG6-OsDES1, and LOG-OsDES1. *DEFECTIVE EMBRYO SAC1* (*OsDES1*) encodes a putative nuclear envelope membrane protein (NEMP)-domain-containing protein. Its mutant *des1* displayed a significant reduction in seed-setting rate due to abortive embryo sac and defective fertilization (Hu et al. 2023), somewhat resembling *Osnrfg6*. *LOG* encodes a cytokinin-activating enzyme required for ovule initiation and pistil development (Kurakawa et al. 2007; Yamaki et al. 2011), which can interact with OsDES1 (Hu et al. 2023). Taken together, OsNRFG6 may form homodimers and form protein complexes with LOG and OsDES1 to participate in regulation of female reproduction and fertilization of NTR lines.

## Conclusions

*OsNRFG6* demonstrates a constitutive expression profile, and encodes a perinucleus and ER-localized protein. The *Osnrfg6* exhibited defective embryo sac development and fertilization, resulting in low seed setting in neo-tetraploid rice. Mutation of *OsNRFG6* led to down-regulation of key reproductive genes during fertilization. OsNRFG6 protein can form homodimers and participate in multi-protein complexes involving LOG and OsDES1. These findings underscore the crucial involvement of *OsNRFG6* in maintaining embryo sac fertility and seed production in neo-tetraploid rice, thereby offering insights into the molecular breeding of polyploid rice.

## Methods

### Plant Materials

*Osnrfg6* was a mutant in neo-tetraploid rice (*Oryza sativa* L.) Huaduo 1 (H1) background, which constructed by CRISPR/Cas9 in our previous study (Yu et al. 2020). H1, a neo-tetraploid rice line registered for Protection for New Varieties of Plants in China in 2016, was developed from the combination of Jackson-4x and 96025-4x (Wu et al. 2020). The pro*OsNRFG6*-GUS transgenic plants and complementary transgenic lines (Com-1 and Com-2) were generated in H1 and *Osnrfg6-2* background, respectively. All materials were cultivated at experimental station of South China Agricultural University, Guangdong, China.

### Phylogenetic Tree Construction about OsNRFG6

The conserved domain of OsNRFG6 protein was analyzed by NCBI Conserved Domain Search (https://www.ncbi.nlm.nih.gov/Structure/cdd/wrpsb.cgi). The full-length protein sequences of 16 homologs were obtained from the NCBI database https://www.ncbi.nlm.nih.gov/). MEGAX was used for multiple alignments (ClustalW method) and phylogenetic tree construction (the neighbor-joining method, bootstrap = 1000) (Kumar et al. 2018).

### Subcellular Localization Analysis

To determine the subcellular localization of OsNRFG6 protein, the coding sequence of *OsNRFG6* was cloned into the pBIN19-GFP vector with the CaMV *35 S* promoter (*Cauliflower mosaic virus*). H2B-mCherry was used as nuclear localization marker (Zhuang et al. 2021). The endoplasmic reticulum (ER) maker was constructed by fusing mCherry with the HDEL ER retention signal (Yuan et al. 2022). The recombinant construct plasmid was transformed into *Agrobacterium strain* (GV3101 strain) and injected into about 5-week-old *Nicotiana benthamiana* leaves. At 3 days after infiltration, the transfected leaves were collected for green fluorescent signals observation under a confocal laser-scanning microscope (Leica DM 2500, Germany, 488 nm laser).

### RT-qPCR Analysis

Total RNAs were isolated using TRIzol reagent (AG, China) followed by treatment with 5× gDNA Clean Reaction Mix to digest genomic DNA (AG, China). About 750 ng RNA from each sample went through reverse transcription to obtain first-strand cDNA (AG, China). RT-qPCR assays were performed using the Hieff qPCR SYBR Green Master Mix (Yeasen, China) and the LightCycler 480II (Roche, Switzerland) system according to the manufacturer’s instructions. The rice *Cytochrome b5* gene (*LOC_Os05g01820.1*) was used as the internal control (Wu et al. 2021). Each measurement was determined for three biological and three technological replications. The relative expression analysis of RT-qPCR was calculated by the 2^−∆∆CT^ method (Livak et al. 2001). All primers used are listed in Table S4.

### GUS Histochemical Staining

A 2 kb genomic sequence before the *OsNRFG6* start codon was amplified via primers (pOsNRFG6-F and pOsNRFG6-R) to be cloned into pCAMBIA1305.1 vector with GUS reporter to generate *proOsNRFG6::GUS* vector. The EHA105 *Agrobacterium tumefaciens* harboring *proOsNRFG6::GUS* was transformed to rice calli for creating transgenic lines, from which developing spikelets in T_1_ generation were collected for GUS histochemical staining according to the manufacturer’s instructions (Leagene, China).

### Cytological Observations

Mature pollen grains were collected before anthesis and stained with 1% I_2_-KI solution, 1% 2, 3, 5-Triphenyte-trazoliumchloride (TTC) solution, optical brightener, and auramine O solution to observe pollen fertility, pollen viability, pollen intine, and exine, respectively. Three spikelets of each plant and three plants of each material were collected, observed and photographed via a Motic BA210 microscope or a fluorescence microscope (Leica DM RXA, Germany).

The whole-mount eosin B-staining confocal laser scanning microscopy (WE-CLSM) observation was performed as follow: The developing or fertilized spikelets were fixed in FAA solution for 24 h. The anthers and ovaries were isolated and hydrated in gradient ethanol (30%, 10%, and 0%) for 30 min per time. After that, the anthers and ovaries were pretreated in 2% aluminum potassium sulfate dodecahydrate for 30 min before being stained with 10–20 mg/L eosin B solution (dissolved with 4% sucrose solution) for 12 h. The samples were rinsed with 2% aluminum potassium sulfate and water (three times), and dehydrated with gradient ethanol solutions (30%, 50%, 70%, 90%, 100%) for 30 min per treatment. Subsequently, the dehydrated samples were hyalinized via 50% (dissolved with ethanol, v/v) and pure methyl salicylate for 2 h and 1 h, respectively. The transparent samples were observed and photographed using a confocal laser-scanning microscope (Leica DM 2500, Germany). Here, the “embryo sac fertility” was used to stand the ratio of normal embryo sac numbers during cytological observation. Each final picture was merged by one to forty optically-sectioned images.

### RNA-seq Analysis

The ovaries at mature embryo sac stage and 1 day after flowering stage were collected in 3 biological replicates (each sample) and stored at -80 ℃ for RNA-seq analysis. The RNA-seq data was obtained using an Illumina NovaSeq6000 system, which was further analyzed via BMKCloud platform (BioMarker Biotech Co., Ltd. Chain). Nipponbare genome (MSU7.0) was used as reference genome (Ouyang et al. 2007). Differentially expressed genes (DEGs) were identified via the criterion with fold change > 1.5 and *p-*value < 0.05. Heat map diagram and Venn analyses were performed by TBtools-II (Chen et al. 2023).

### Yeast Two-Hybrid Assay

The truncated and full-length *OsNRFG6* CDS were cloned into the pGBKT7 or pGADT7 vector to generate BD-OsNRFG6^BS^ (36–308 aa), BD-OsNRFG6^PAN^ (325–458 aa), BD-OsNRFG6^C^ (482–813 aa), BD-OsNRFG6, AD-OsNRFG6^BS^, AD-OsNRFG6^PAN^, AD-OsNRFG6^C^ and AD-OsNRFG6 vectors, respectively. The recombinant AD constructs were transformed with recombinant BD constructs into yeast strain Y2H Gold, which were screened on SD/-Trp-Leu medium, and cultured on SD/-Trp-Leu-His-Ade medium (30 ℃) to test the protein interactions.

### Bimolecular Fluorescence Complementation Assay (BiFC)

The CDS of *OsNRFG6* (*LOC_Os06g40030.1*), *LOG* (*LOC_Os01g40630.1*) and *OsDES1* (*LOC_Os03g31570.1*) were fused with cYFP or nYFP in p2YN/C system to generate nYFP-OsNRFG6, cYFP-OsNRFG6, nYFP-LOG, cYFP-LOG, and cYFP-OsDES1 vectors, respectively (Zhuang et al. 2021). Subsequently, the recombinant constructs were transformed *Agrobacterium* strain (GV3101 strain) and co-infected into *Nicotiana benthamiana* leaves. At 3–5 days post infection, the infected *Nicotiana benthamiana* leaves were collected for fluorescence observation under a confocal laser-scanning microscope (Leica DM 2500, Germany).

### Luciferase Complementation Imaging Assay (LCI)

For the luciferase complementation imaging assay, the *OsNRFG6* CDS, its truncated CDS (PAN fragment), *LOG* CDS, and *OsDES1* CDS were fused with nLUC or cLUC in pCAMBIA1300 to construct nLUC-OsNRFG6, cLUC-OsNRFG6, nLUC-OsNRFG6^PAN^, cLUC-OsNRFG6^PAN^, cLUC-LOG, and nLUC-OsDES1 vectors, respectively. The recombined nLUC and cLUC vectors were coupled and co-transformed into *Nicotiana benthamiana* leaves via GV3101 *Agrobacterium* strain. The leaves were sprayed with 1 mM D-Luciferin potassium salt (Promega, P1043, USA) and observed under CCD imaging apparatus (Berthold, NightSHADE LB985, Germany) at least 72 h post infiltration.

### Electronic Supplementary Material

Below is the link to the electronic supplementary material.

Supplementary Material 1

Video S1. Normal embryo sac in H1.

Video S2. Abnormal position of polar nuclei in Osnrfg6.

Supplementary Material 4

Video S3. Egg apparatus degeneration in Osnrfg6.

Video S4. Female germ unit degeneration in Osnrfg6.

## Data Availability

The raw reads of RNA-seq were deposited in at the NGDC BIG Submission with accession ID PRJCA025224. The sequences and annotations of rice *japonica* reference genome MSU7 are available from the website http://rice.plantbiology.msu.edu/. All data supporting the conclusions described here are provided in tables, figures, and additional files.

## Abbreviations

ATR: autotetraploid rice
PMeS: Polyploid Meiosis Stability
NTR: neo-tetraploid rice
OsNRFG6: NEO-TETRAPLOID RICE FERTILITY RELATED GENE 6
LecRLKs: Lectin receptor-like kinases
RT-qPCR: transcription quantitative real-time PCR
WE-CLSM: whole-mount eosin B-staining confocal laser scanning microscopy
Y2H: yeast two-hybrid
LCI: luciferase complementation imaging
BiFC: bimolecular fluorescence complementation
DEGs: differentially expressed genes
KEGG: Kyoto Encyclopedia of Genes and Genomes
