# Supplementary material for: Lectin Receptor-Like Protein Kinase OsNRFG6 is Required for Embryo Sac Development and Fertilization in Neo-Tetraploid Rice
Source: Rice (N Y). 2024 Jun 25;17:41. doi: 10.1186/s12284-024-00720-0 (PMC11199475; doi:10.1186/s12284-024-00720-0)
Supplement: Supplementary file 4 — Supplementary Material 4 [file 12284_2024_720_MOESM4_ESM.docx]

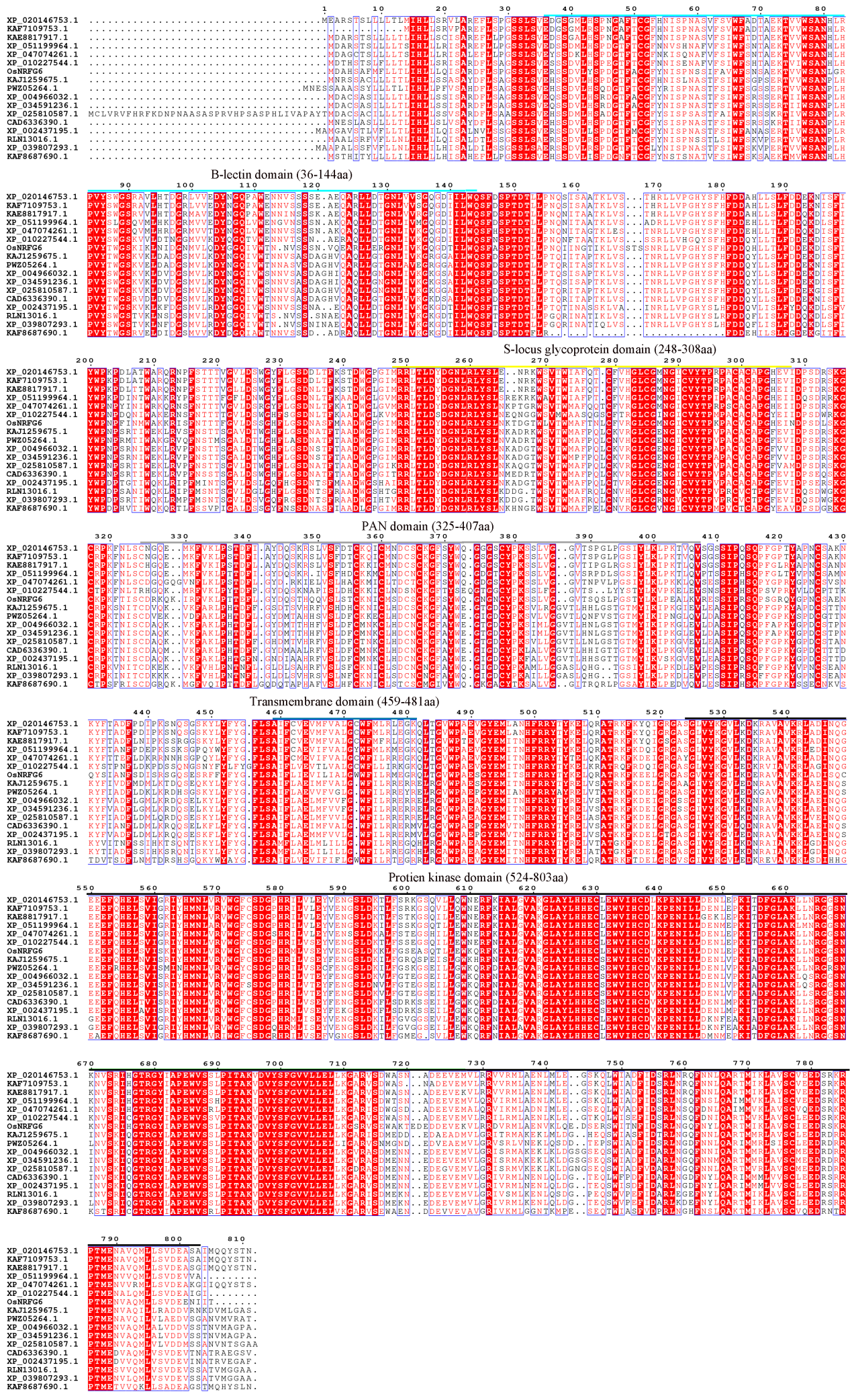


**Fig. S1. Protein sequence alignment of OsNRFG6 and its homologs from 16 different species.**

Protein sequence alignment was performed among OsNRFG6 and its homologs from *Aegilops tauschii subsp* (XP_020146753.1), *Triticum aestivum* (KAF7109753.1), *Hordeum vulgare* (KAE8817917.1), *Lolium perenne* (XP_051199964.1), *Lolium rigidum* (XP_047074261.1), *Brachypodium distachyon* (XP_010227544.1), *Digitaria exilis* (KAF8687690.1), *Panicum miliaceum* (RLN13016.1), *Panicum virgatum* (XP_039807293.1), *Miscanthus lutarioriparius* (CAD6336390.1), *Sorghum bicolor*(XP_002437195.1), *Panicum hallii* (XP_025810587.1), *Setaria italica* (XP_004966032.1), *Setaria viridis* (XP_034591236.1), *Paspalum vaginatum* (KAJ1259675.1), and *Zea mays* (PWZ05264.1).

**
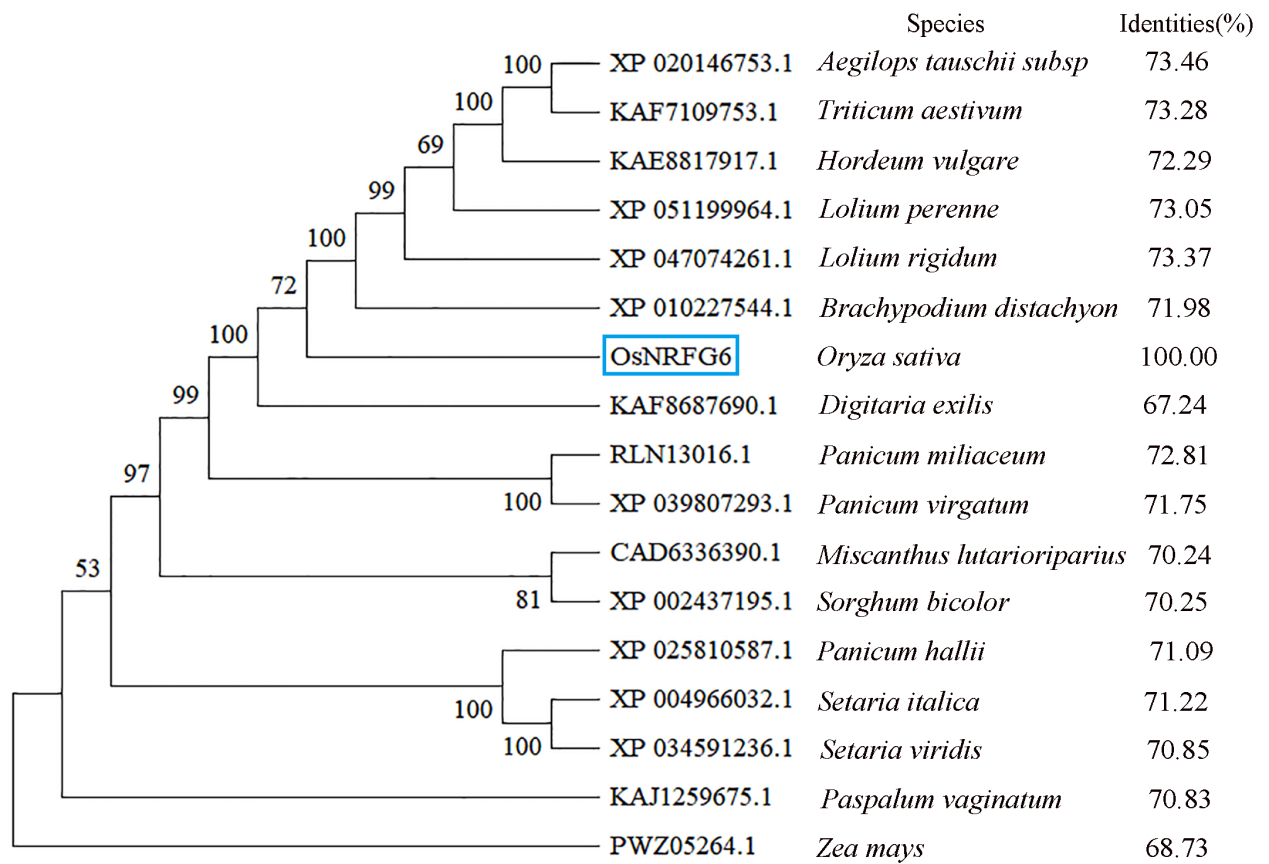
**

**Fig. S2. Phylogenetic analysis of OsNRFG6.**


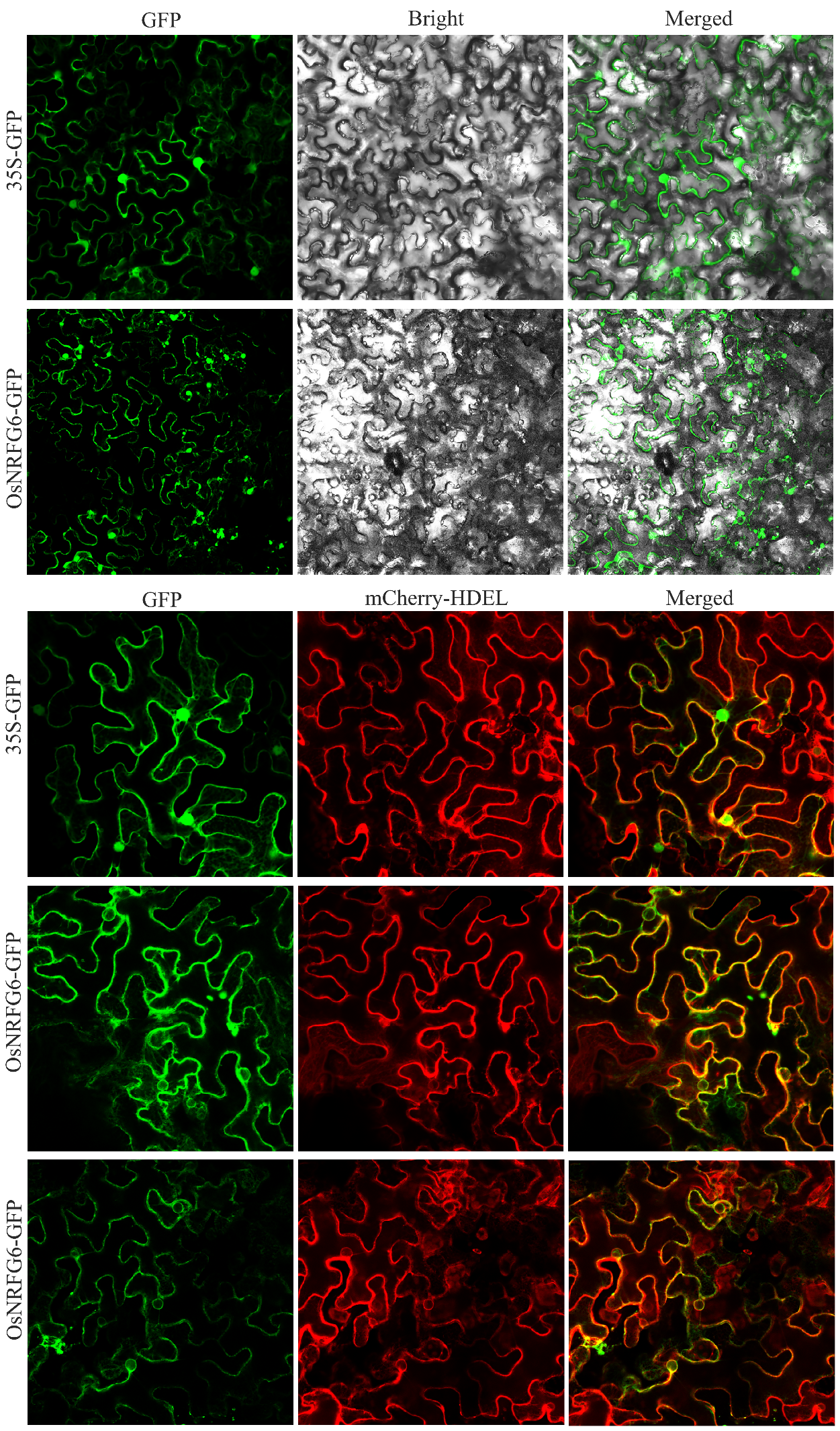


**Fig. S3. Subcellular localization of OsNRFG6.**

Subcellular localization of OsNRFG6-GFP fusion proteins in *Nicotiana benthamiana* leaf epidermal cells. mCherry-HDEL indicates an endoplasmic reticulum marker. 35S-GFP was used as the control.


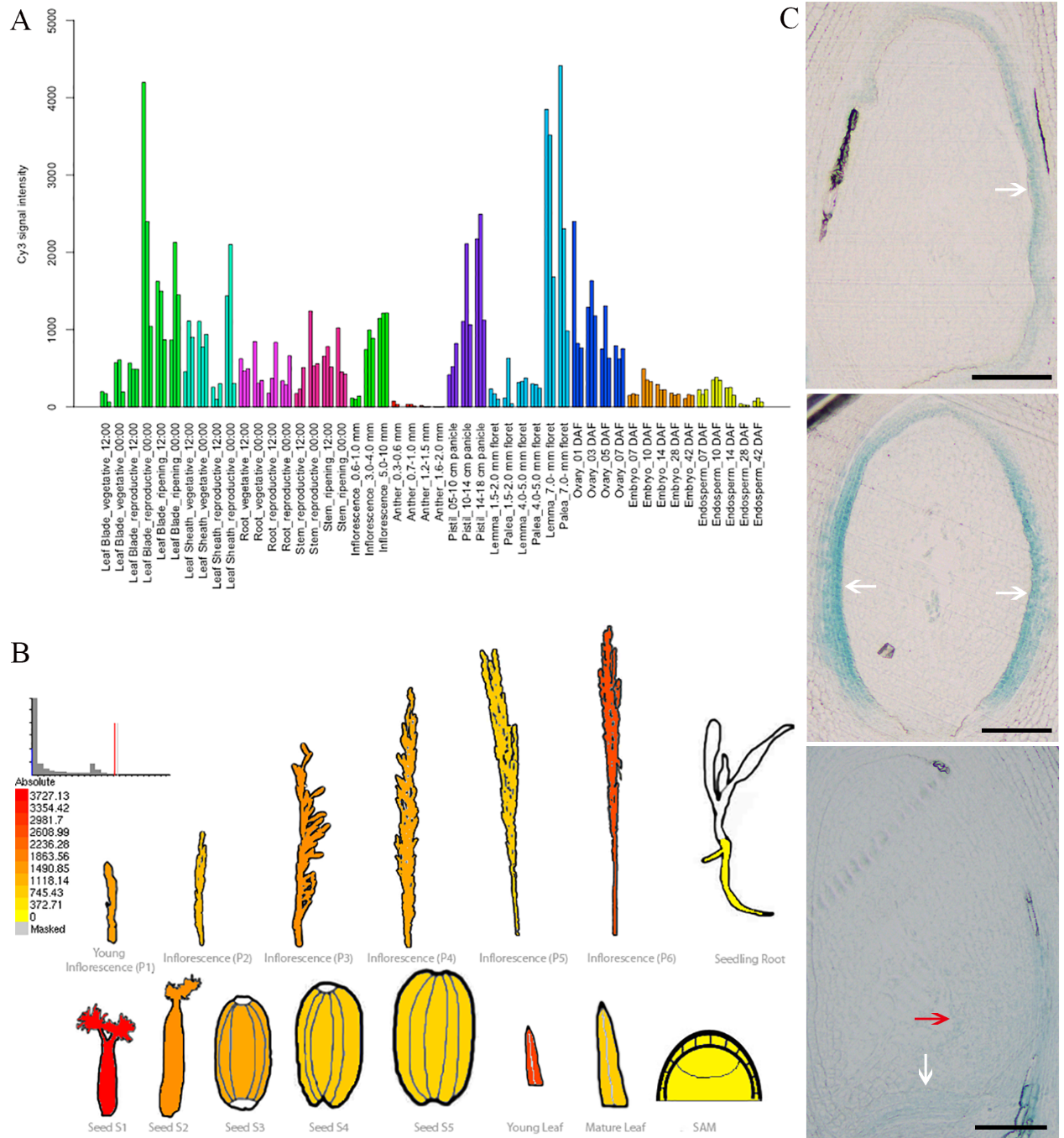


**Fig. S4.** **The expression pattern analysis of *OsNRFG6* using the RiceXPro database, Rice eFP Browser database, and GUS reporter system.**

(A) The expression pattern analysis of *OsNRFG6* in RiceXPro database. (B) The expression pattern analysis of *OsNRFG6* in Rice eFP Browser database. (C) Semi-thin sections of the embryo sacs by GUS histochemical staining in H1. The white arrows indicate the ovule wall and the red arrow indicates cavity of ovule. Scales bar = 1 μm.


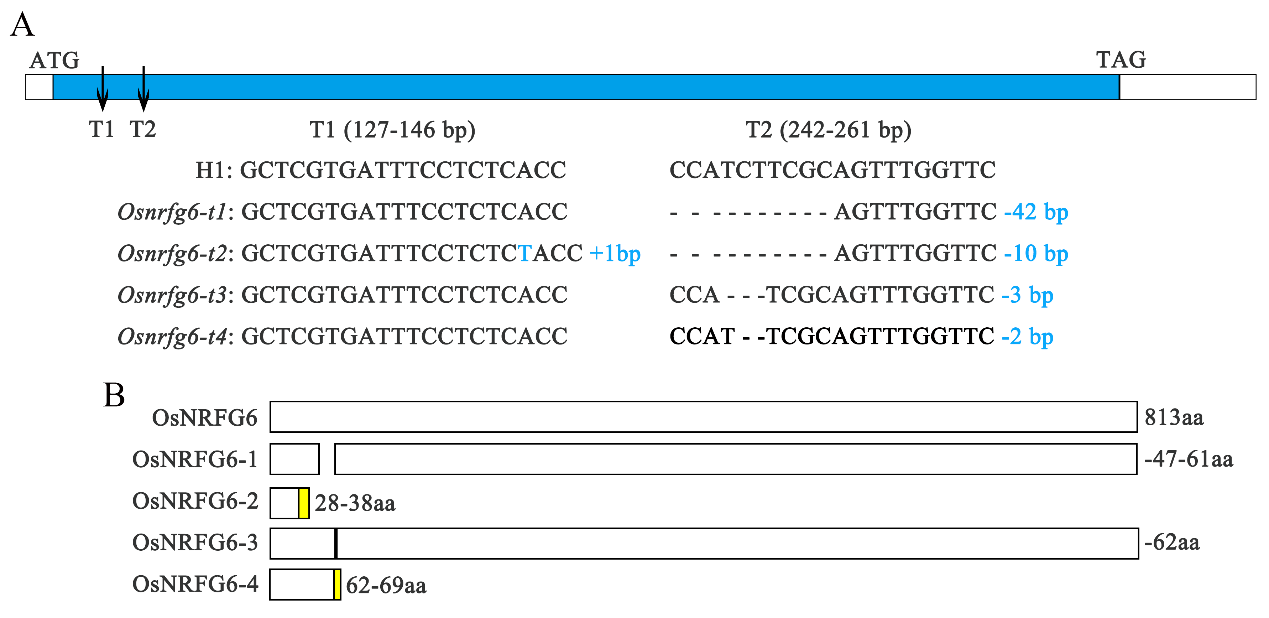


**Fig. S5. Schematic diagram of CRISPR/Cas9 target sites for *OsNRFG6*.**

(A) Gene structure of *OsNRFG6* and nucleotide sequences of the CRISPR/Cas9 target sites in H1 and *Osnrfg6* mutants. Blue box indicates the exon. White boxes indicate the untranslated regions. (B) Predicted translated protein residues in H1 and *Osnrfg6* mutants.


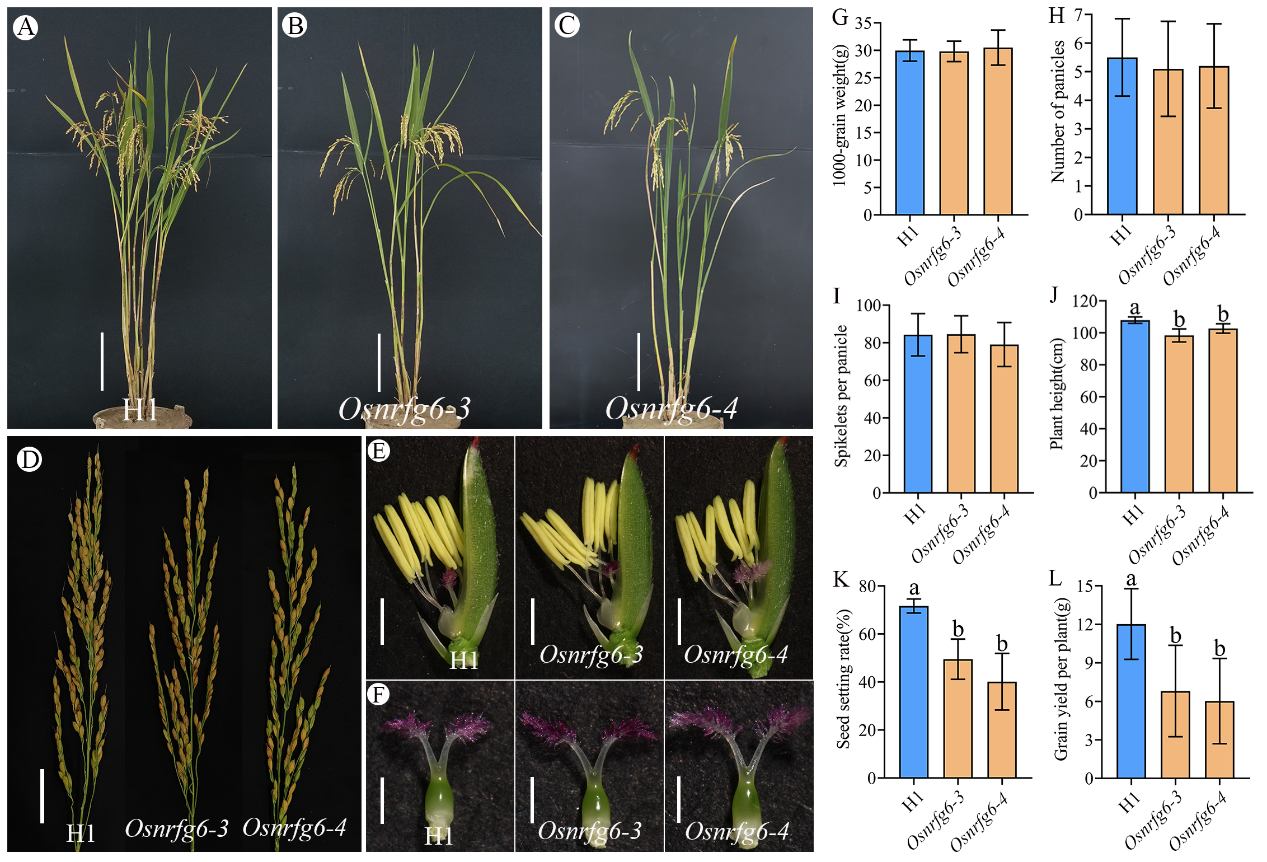


**Fig. S6. Phenotypic characterization of *Osnrfg6*.**

(A-C) Plants morphology, (D) mature panicles, (E) mature anthers, (F) mature pistils, (G) 1000-grain-weight, (H) number of panicles, (I) spikletes per panicle, (J) plant height, (K) seed-setting rate, and (L) grain yield per plant in H1, *Osnrfg6-3* and *Osnrfg6-4*, respectively. Scale bar = 20 cm (A-C), 4 cm (D), 2 mm (E). Error bars indicate the SD with *n* = 10. Different letters indicate significant differences (*P* < 0.01, Least signiﬁcant diﬀerence).


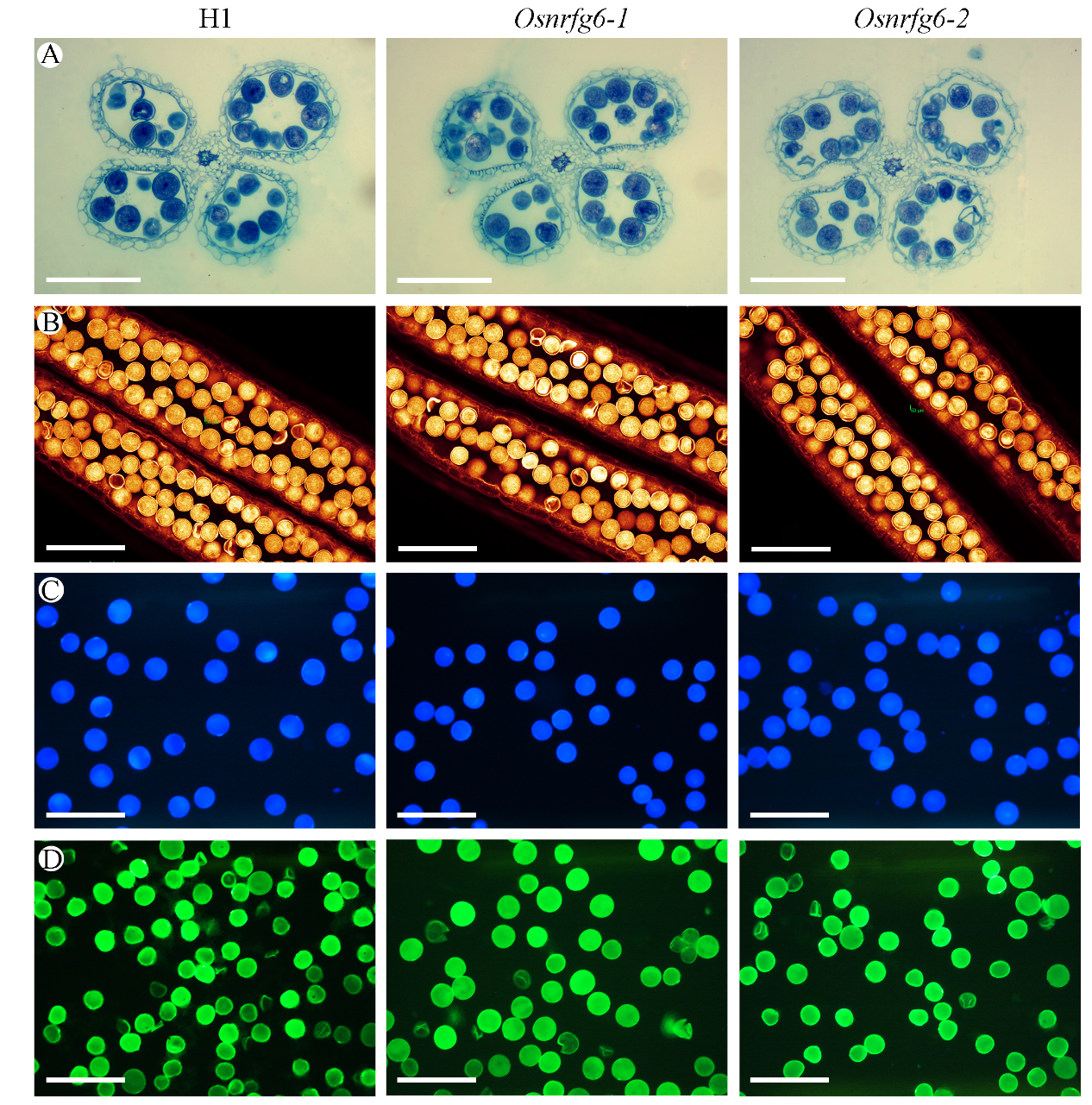


**Fig. S7. Cytological observation of mature pollen grains in H1 and *Osnrfg6*.**

(A) A semi-thin sectioning observation of mature pollen anthers, (B) mature pollen grains stained with eosin B, (C) mature pollen grains stained with fluorescent brightener, (D) mature pollen grains stained with auramine O in H1, *Osnrfg6-1* and *Osnrfg6-2*. Scale bars = 200 μm (A, C, D), and 50 μm (B).


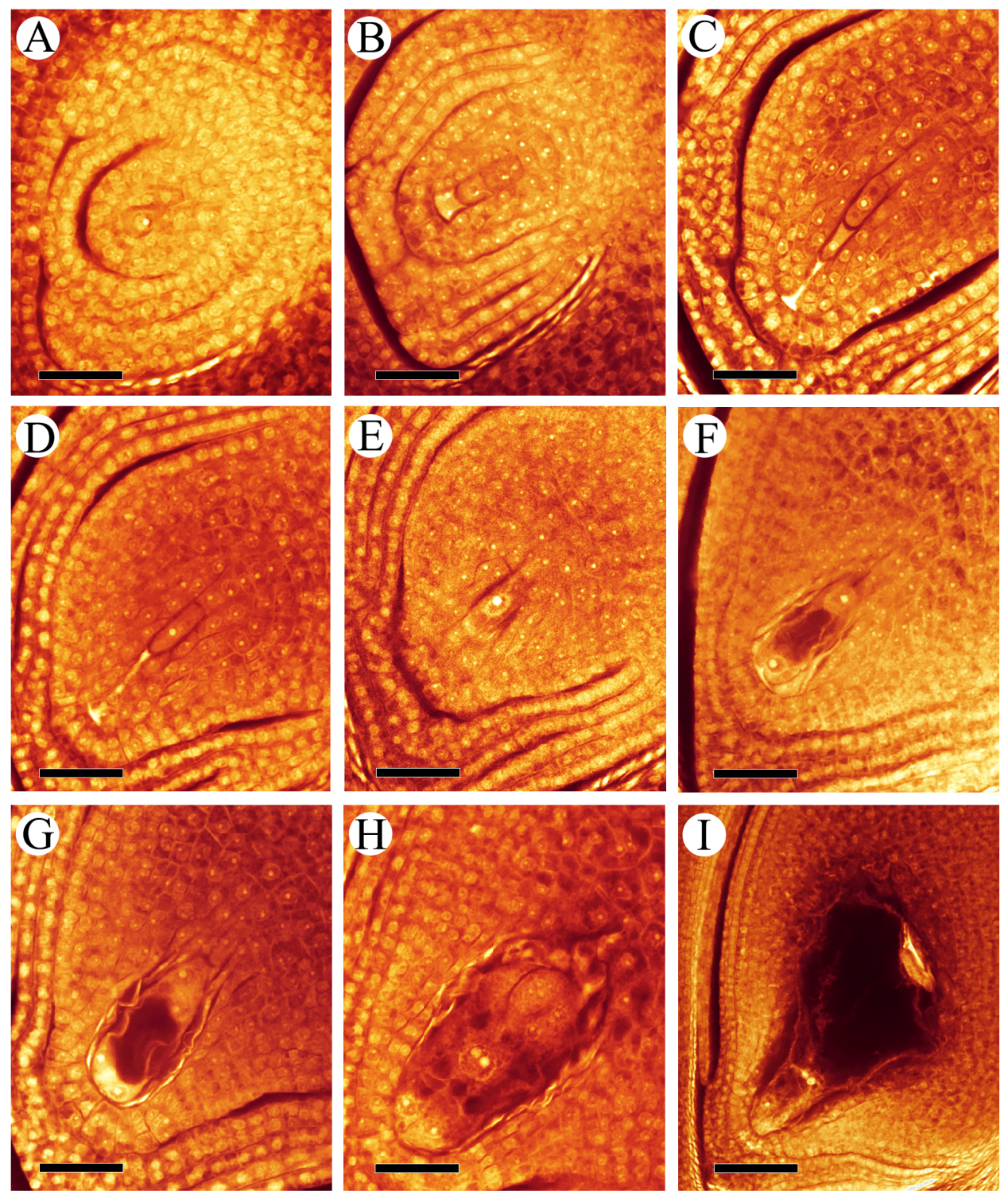


**Fig. S8. WE-CLSM observation of the embryo sac development in H1.**

(A) Megasporocyte. (B) Tetrad. (C) Functional megaspore formation. (D) Functional megaspore. (E) Mono-nucleate embryo sac. (F) Two-nucleate embryo sac. (G) Four-nucleate embryo sac. (H) Middle eight-nucleate embryo sac. (I) Mature embryo sac. Scale bars = 80 μm.


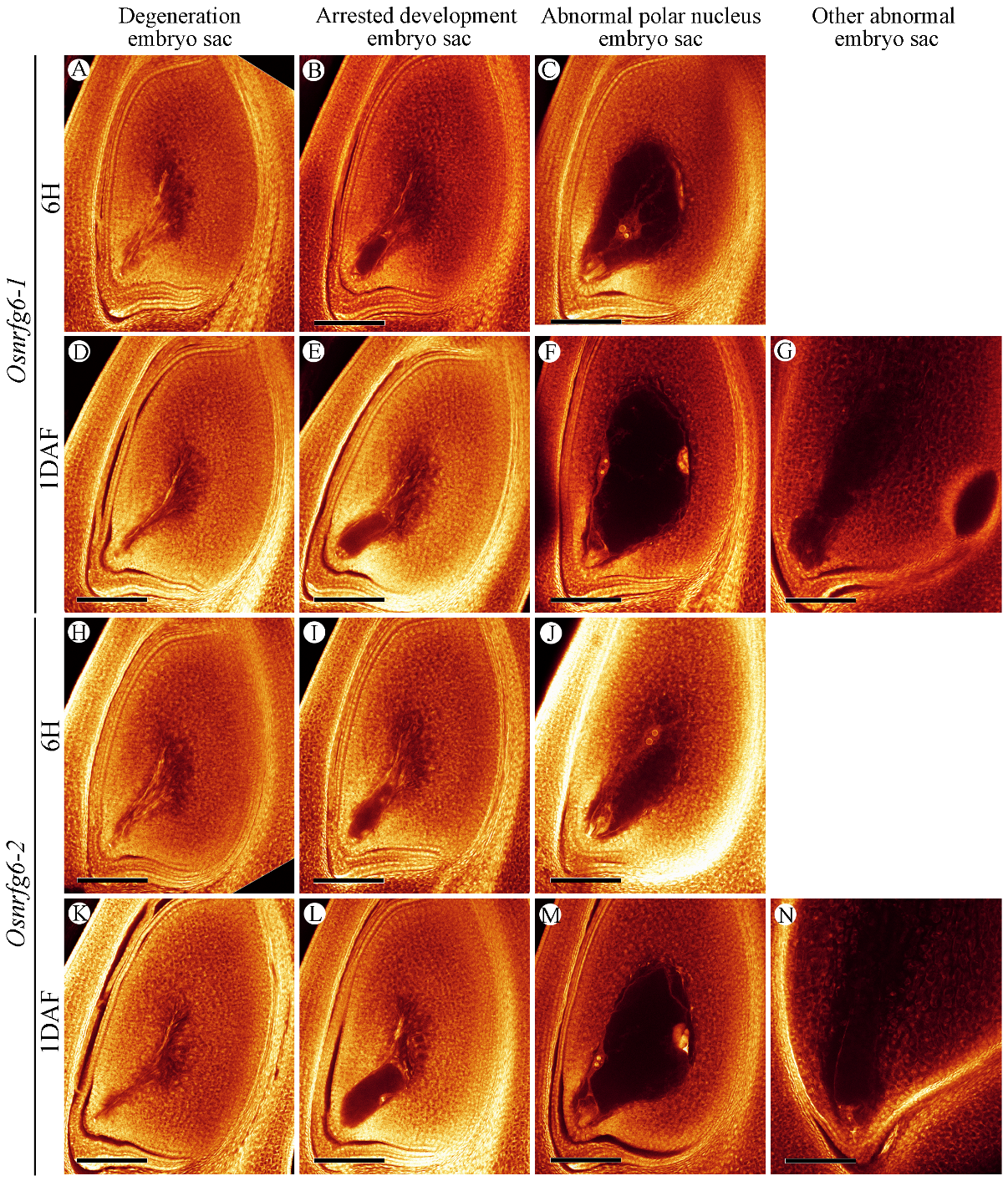


**Fig. S9. WE-CLSM observation of abnormal double fertilization and embryogenesis development in *Osnrfg6*.**

(A-C, H-J) Degeneration embryo sacs, arrested development embryo sacs and abnormal position of polar nuclei embryo sacs in *Osnrfg6* at 6 hours after flowering. (D-G, K-N) Degeneration embryo sacs, arrested development embryo sacs, abnormal position of polar nuclei embryo sacs and other abnormal embryo sacs in *Osnrfg6* at 1 day after flowering. Scale bars=150 μm. 6H, 6 hours after flowering; DAF, day after flowering.


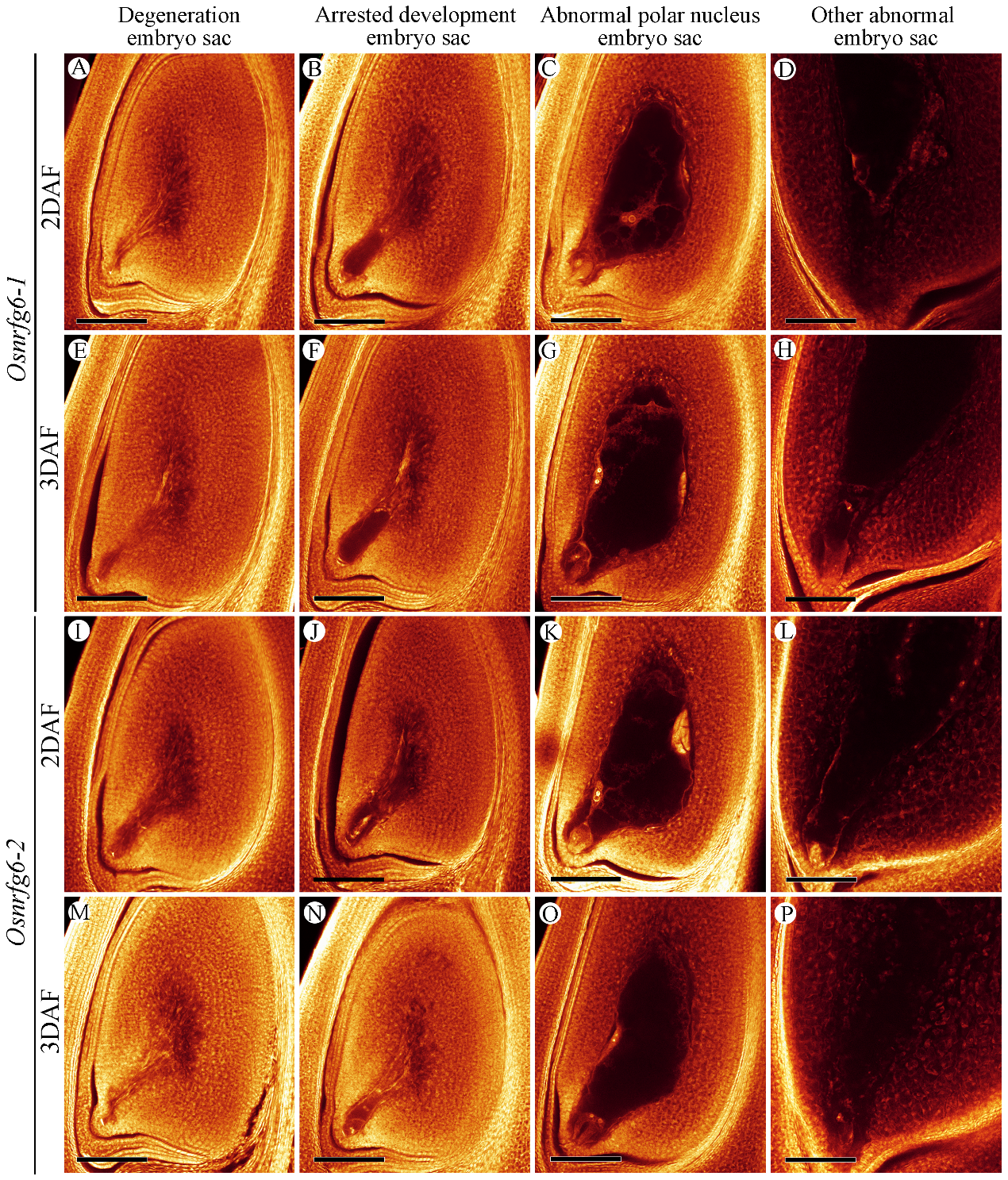


**Fig. S10.** **WE-CLSM observation of abnormal embryogenesis development in *Osnrfg6*.**

(A-D, I-L) Degeneration embryo sacs, arrested development embryo sacs, abnormal position of polar nuclei embryo sacs and other abnormal embryo sacs in *Osnrfg6* at 2 days after flowering. (E-H, M-P) Degeneration embryo sacs, arrested development embryo sacs, abnormal position of polar nuclei embryo sacs and other abnormal embryo sacs in *Osnrfg6* at 3 days after flowering. Scale bars = 150 μm. DAF, days after flowering.


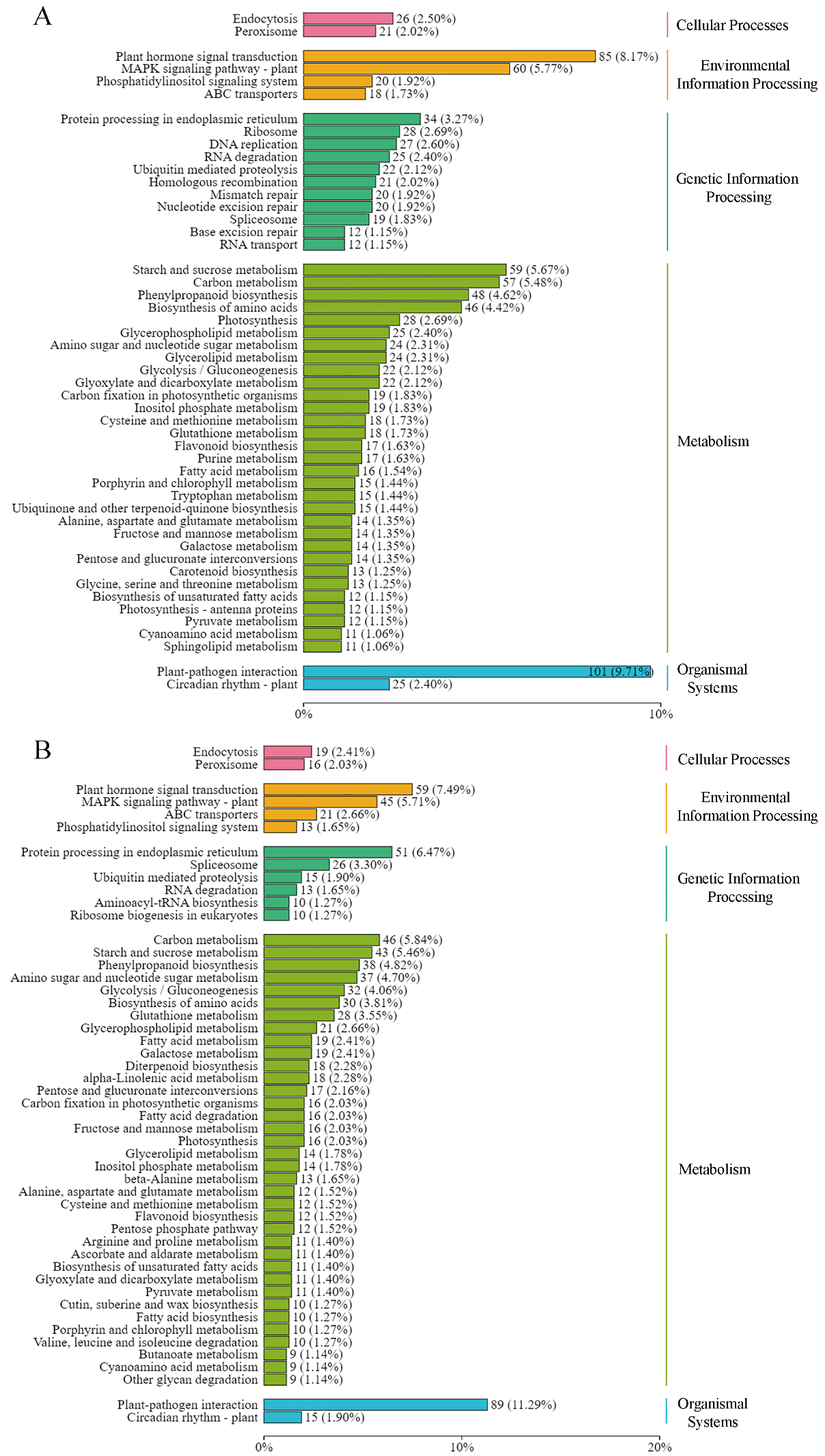


**Fig. S11. Kyoto Encyclopedia of Genes and Genomes (KEGG) pathway analysis of DEGs in H1 and *Osnrfg6*.**

(A) KEGG pathway analysis of DEGs in H1 and *Osnrfg6* at mature embryo sac stage. (B) KEGG pathway analysis of DEGs in H1 and *Osnrfg6* at 1 day after flowering stage.


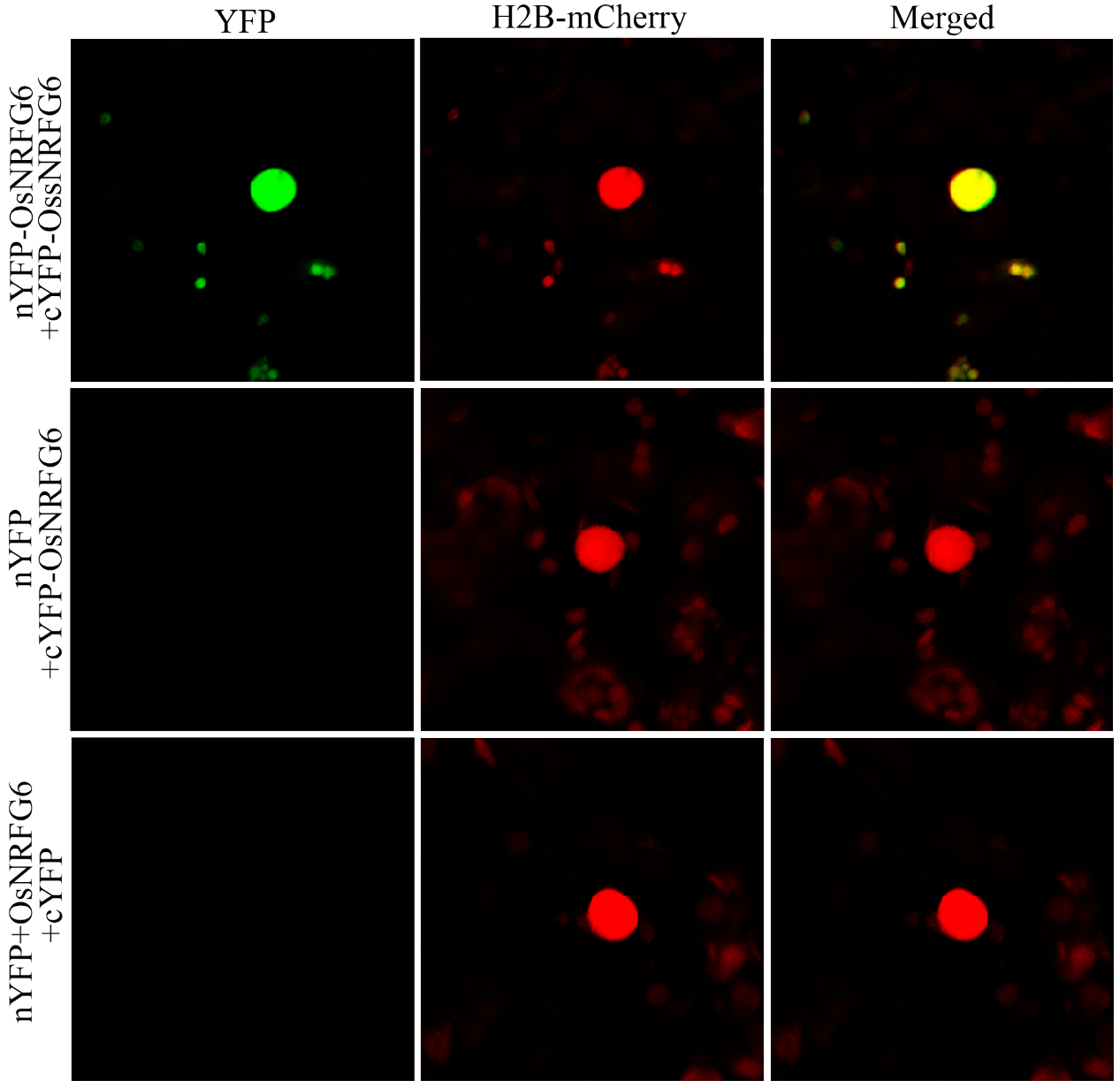


**Fig. S12. OsNRFG6 interacted with itself by the BiFC assay performed in *Nicotiana benthamiana* leaf epidermal cells.**
